# Comparative study of the digestion and metabolism related genes’ expression changes during the postnatal food change in different dietary mammals

**DOI:** 10.3389/fgene.2023.1198977

**Published:** 2023-07-04

**Authors:** Zhuma Yizhen, Lei Chen, Xiaodie Jie, Fujun Shen, Liang Zhang, Yusen Hou, Lu Li, Guoqiang Yan, Xiuyue Zhang, Zhisong Yang

**Affiliations:** ^1^ Sichuan Academy of Giant Panda, Chengdu, China; ^2^ Sichuan Key Laboratory of Conservation Biology on Endangered Wildlife, College of Life Sciences, Sichuan University, Chengdu, China; ^3^ Sichuan Key Laboratory for Conservation Biology of Endangered Wildlife, Chengdu Research Base of Giant Panda Breeding, Chengdu, China; ^4^ Pharmacy College, Chengdu University of Traditional Chinese Medicine, Chengdu, Sichuan, China

**Keywords:** giant panda, red panda, transcriptome, feeding habit, adaptive response

## Abstract

The changes in the expression of genes related to digestion and metabolism may be various in different dietary mammals from juvenile to adult, especially, the giant panda (*Ailuropoda melanoleuca*) and red panda (*Ailurus fulgens*), which were once carnivores but have shifted to being specialized bamboo eaters, are unique features of their changes are more unclear. To elucidate the changing patterns of gene expression related to digestion and metabolism from juvenile to adult in different dietary mammals, we performed transcriptome analysis of the liver or pancreas in giant and red pandas, herbivorous rabbits (*Oryctolagus cuniculus*) and macaques (*Macaca mulatta*), carnivorous ferrets (*Mustela putorius furo*), and omnivorous mice (*Mus musculus*) from juvenile to adult. During the transition from juvenile to adulthood, giant and red pandas, as well as rabbits and macaques, show significant upregulation of key genes for carbohydrate metabolism, such as starch hydrolysis and sucrose metabolism, and unsaturated fatty acid metabolism, such as linoleic acid, while there is no significant difference in the expression of key genes for fatty acid β-oxidation. A large number of amino acid metabolism related genes were upregulated in adult rabbits and macaques compared to juveniles. While adult giant and red pandas mainly showed upregulation of key genes for arginine synthesis and downregulation of key genes for arginine and lysine degradation. In adult stages, mouse had significantly higher expression patterns in key genes for starch hydrolysis and sucrose metabolism, as well as lipid and protein metabolism. In contrast to general expectations, genes related to lipid, amino acid and protein metabolism were significantly higher expressed in adult group of ferrets, which may be related to their high metabolic levels. Our study elucidates the pattern of changes in the expression of genes related to digestion and metabolism from juvenile to adult in different dietary mammals, with giant and red pandas showing adaptations associated with specific nutritional limitations of bamboo.

## 1 Introduction

Food not only supports the continuation of animal life but is also an important driver of animal evolution (Jaenisch and Bird, 2003; Lillycrop et al., 2007; Mathieson and Mathieson, 2018; Harris et al., 2019; Mathieson, 2020; Rees et al., 2020). Mammals feed on breast milk as juveniles and have a diverse diet as adults, but they can be approximately classified as carnivorous, omnivorous, and herbivorous. In any case, mammals undergo a large change in feeding habits from juvenile to adulthood, and how the expression of genes related to digestion and metabolism changes in response to this change in feeding habits and the mechanisms of the changes have not been well elucidated.

Although giant panda (*Ailuropoda melanoleuca*) and red panda (*Ailurus fulgens*) both are carnivores, they shifted to feeding on bamboo about the late Miocene (around 4.5 Mya) (Zhao et al., 2010; Hu et al., 2017). Bamboo is an exceptional food, with high fiber and low fat as well as a deficiency of some essential nutrients such as lysine (Hu et al., 2017). In addition, bamboo is rich in plant secondary metabolites (Zhu et al., 2018). After shifting from a meat diet to a specialized bamboo diet, giant and red pandas have evolved many adaptive physiological features, such as well-developed jaw muscles and molars (Figueirido et al., 2012), pseudogenization of the fresh taste receptor gene (T1R1) on the genome of giant and red pandas (Li et al., 2010), the six-finger structure of giant and red pandas to facilitate grasping bamboo stems (Hu et al., 2017), and adaptive evolution of intestinal flora (Zhu et al., 2018). In any case, giant and red pandas still retain carnivore digestive features, such as having a simple digestive system and not evolving a herbivore-specific digestive tract such as the ruminant stomach and cecum (Li et al., 2010; Hu et al., 2017; McKenney et al., 2018; Zhu et al., 2018). The genomes still retain carnivore-specific genetic features, with complete genes encoding lipases, proteases, etc. (Li et al., 2010). Mammals with different diets have diets that change from suckling to adult carnivory, herbivory, and omnivory, as well as from ancestral carnivory to the current specialized specialization of herbivory like giant pandas and red pandas. It is unclear what the characteristics of the altered expression of each of their digestion and metabolism related genes are.

The liver and pancreas are important organs for digestion and metabolism. In this study, we performed transcriptome sequencing (RNA-seq) of liver and pancreas tissues of giant pandas, red pandas, and ferrets (*Mustela putorius* furo) at three different growth and developmental stages and combined RNA-seq data from liver samples of rabbits (*Oryctolagus cuniculus*), macaques (*Macaca mulatta*), and mice (*Mus musculus*) at three developmental stages to profile gene expression patterns in the liver or pancreas of different dietary species at different growth stages. And further elucidate the expression change patterns of digestion and metabolism related genes during growth and development to explore the adaptive responses of giant and red pandas to low-nutrient bamboo diet.

## 2 Materials and methods

### 2.1 Sample collection

In this study, fifty-six liver samples and twenty-five pancreas samples were collected from bamboo-eating giant pandas and red pandas, herbivore rabbits and macaques, carnivore ferrets, and omnivore mice. All samples were grouped according to species’ developmental and dietary stages into no feeding, suckling and adult stages, as shown in Supplementary Table S1. Giant panda tissue samples and red panda tissue samples were provided by the Chengdu Research Base of Giant Panda Breeding and the Wolong Giant Panda Research and Conservation Center at Dujiangyan, Sichuan Province, China. All individuals died naturally or accidently and were not related to the design of this experiment or the organs sampled. All ferret tissue samples were obtained from Wuxi Kuboyi Pet Products Co., Ltd. (Wuxi, China). The ferrets were anesthetized and euthanized, and their liver and pancreas samples were dissected in our laboratory. The remaining RNA-seq data for liver from rabbits, macaques, and mice were downloaded from the NCBI Short Read Archive (SRA) (Supplementary Table S1). All samples described in this study were approved by the Ethics Committee of the College of Life Sciences at Sichuan University (approval number: 20190506001). The liver and pancreas sampled were normal in morphology and size, and none of them showed pathological changes. Freshly excised liver and pancreas tissue samples were immediately stored at −80°C.

### 2.2 Extraction of total RNA

Before RNA extraction, the liver and pancreas samples we collected were dissected and homogenized. Total RNA was extracted from liver and pancreas samples using the TRIzol reagent (Invitrogen, Carlsbad, CA, United States) and treated with RNase-free DNase. Samples with RNA integrity numbers (RINs) greater than 5.8 were allowed for the next step of RNA-seq library construction.

### 2.3 Library preparation and sequencing

Sequencing libraries were prepared using the NEBNext^®^ Ultra™ RNA Library Preparation Kit for Illumina^®^ (NEB, United States) following the manufacturer’s recommendations, and index codes were added to the attribute sequences of each sample. After column purification, the quality of the obtained libraries was assessed on an Agilent Bioanalyzer 2100 system. Libraries are sequenced on the Illumina Hiseq 2000 platform. These reads are available from the NCBI Sequence Read Archive, BioProject Accession Number PRJNA892217 and PRJNA612421.

### 2.4 Quality control and alignment of raw sequencing data

For red panda, we used the reference genome and reference annotation downloaded from DNA ZOO (https://www.dnazoo.org/). The genome of red panda in DNA ZOO was reassembled using the 3D-DNA pipeline (Dudchenko et al., 2017) and reviewed using Juicebox Assembly Tools (Dudchenko et al., 2018) based on the draft assembly ASM200746v1 (GCA_002007465.1) (Hu et al., 2017). Reference genomes and reference annotations of the other five mammals were downloaded from Ensembl Release 98.

First, low quality reads with quality scores below 20 and adapter sequences were removed from the raw data using NGS QC Toolkit (version 2.3.3) (Patel and Jain, 2012) and assessed by FastQC (version 0.11.9) (http://www.bioinformatics.babraham.ac.uk/projects/fastqc/) to assess the quality of the raw sequencing data. Then, high quality reads that passed the screening threshold were mapped to the reference genome using HISAT2 (version 2.1.0) (Kim et al., 2019). Finally, the alignment files in SAM format were converted to BAM format by SAMtools (version 1.9) (Li et al., 2009), and featureCounts in the Subread package (version 1.6.4) were chosen to estimate the expression abundance of all genes (Liao et al., 2014).

### 2.5 Phylogenetic tree construction, principal component analysis and spearman correlation clustering analysis

To assess the grouping relationships and clustering patterns among all samples from six species, we identified 1:1 single copy orthologous genes in all species and corrected the gene expression levels of different species. Based on the corresponding version numbers of the genomes, we downloaded the protein coding sequences of different species and screened the sequences: i) removing sequences with less than 50 amino acids; ii) removing sequences with early terminators; and iii) keeping the longest protein coding sequences. Subsequently, BLASTp alignment (E value cutoff of 1E-5) was performed using Orthofinder 2.3.7 software (Emms and Kelly, 2015), based on the reciprocal best hit algorithm. A total of 9520 1:1 single copy orthologous genes were identified among six species for further analysis. In addition, we used RAxML (Stamatakis, 2014) software to construct a phylogenetic maximum-likelihood tree of amino acid sequences of orthologous genes by the maximum likelihood (ML) method. When comparing between different species, we need to correct not only for sequencing depth but also for the length of the same gene across species. Therefore, we normalized 1:1 single copy orthologous genes’ expression level using GeTMM (based on gene length correction TMM) (Smid et al., 2018), which combines gene length correction with a trimmed mean of M values (TMM) normalization method to enable comparable expression levels of homologous genes among different species. First, we construct an expression matrix for the samples, with the rows being the expression of the orthologous genes and the columns being the names of the samples. After that, we perform the length correction, calculate the correction factor, and normalize the sequencing depth of the samples. The normalized expressions were filtered to remove genes with zero gene expression in all of the same species. Finally, we performed a log2 transformation of the normalized gene expression matrix for each tissue. The principal component values of each sample were calculated using the prcomp function of the R package stats and visualized using the ggplot function of the R package ggplot2 for graphing (Valero-Mora, 2010). Clustering analysis of Spearman correlations between samples was performed using the cor function of the R package stats, followed by visualization using the heatmap.2 function of the R package gplots.

### 2.6 Identification of differentially expressed genes

For comparative analysis of gene expression change patterns among different developmental stages in each species, we used the filterByExpr function of the R package edgeR to filter the low-expressed genes of each species’ liver or pancreas raw expression matrices separately (Robinson and Oshlack, 2010). By default, the filterByExpr function selects the smallest number of samples within a group as the minimum number of samples, keeping genes with 10 or more sequence fragment counts in at least this number of samples. Since the number of reads for RNA-seq can be affected by technical errors, sequencing depth, and gene length, we normalized the gene reads based on the TMM algorithm in edgeR (Robinson and Oshlack, 2010). And then, we analyzed the gene expression changes between no feeding and suckling stages, suckling and adult stages based on the normalized gene expression matrices of the liver and pancreas for each species. Genes with a Benjamini-Hochberg false discovery rate of 0.05 and |log2FC| ≥ 1 were identified as significantly differentially expressed genes (DEGs).

### 2.7 Enrichment analysis of differentially expressed genes

GO term and KEGG pathway enrichment analyses were performed on the screened DEGs using the enricher function of the R package clusterProfiler (Yu et al., 2012). GO terms for all genes of five species, except for the red panda, were extracted by the highly customizable BioMart data mining tool. While GO terms for all genes of the non-model mammal red panda were annotated by EggNOGv5.0 (http://eggnog5.embl.de/#/app/home). KEGG pathways for each gene of all species were extracted from the KEGG pathway database (https://www.genome.jp/kegg/pathway.html). PvalueCutoff < 0.05 was defined as a significant GO entry or KEGG pathway.

### 2.8 Real-time quantitative PCR

We validated four DEGs associated with liver digestion and metabolism in red pandas and ferrets, respectively. Due to the extreme difficulty in obtaining samples of giant pandas, we have not collected enough samples for RT-qPCR experiments as the preliminary work has run out of samples. Fluorescent quantitative PCR primers were designed using Primier-Blast (https://www.ncbi.nlm.nih.gov/tools/primerblast/index.cgi), and the primer sequences are shown in Supplementary Table S38. Total RNA was extracted using M5 Universal RNA Mini Kit (Mei5 Biotechnology, China) according to the manufacturer’s instructions. The RNA of red pandas, ferrets were used for real-time PCR assay (Supplementary Table S39). The isolated RNA was converted to double-stranded cDNA using M5 Sprint qPCR RT Kit (Mei5 Biotechnology, China).

After the cDNA synthesis, quantification of 10 mRNA levels was conducted by real-time PCR performed on a CFX96 real-Time PCR Detection System. The total volume of 10 μl reaction mix for the real-time PCR contained 5 μl 2X M5 HiPer SYBR Premix EsTaq (with Tli RNaseH) (Mei5 Biotechnology, China), 0.2 μl forward primer (10 pmol/μl), 0.2 μl reverse primer (10 pmol/μl), and 1 μl cDNA severed as a template and 3.6 μl ddH2O. Negative controls containing water as template were also included in each run. The cycling conditions were as follows: 1 cycle of 95 C for 30 s; 40 cycles of 95°C for 5 s, 60°C for 30 s. Then, the expression levels of the mRNAs above were analyzed using the relative quantification (delta-Ct method). The housekeeping gene, GAPDH, was included as internal controls in all RT-qPCR runs. Expression of each gene verified by RT-qPCR were showed in Supplementary Table S40.

## 3 Results

### 3.1 The principal component analysis and clustering analysis of transcriptome data

In this study, we performed a combined analysis on the transcriptome data of pancreas or liver tissues in nine red pandas, nine giant pandas, nine ferrets, eleven rabbits, eight macaques, and twelve mice. The alignment rate of RNA-seq read segments in this study ranges from 81.76% to 97.55%, which is a relatively high alignment rate (Supplementary Table S1).

**FIGURE 1:**
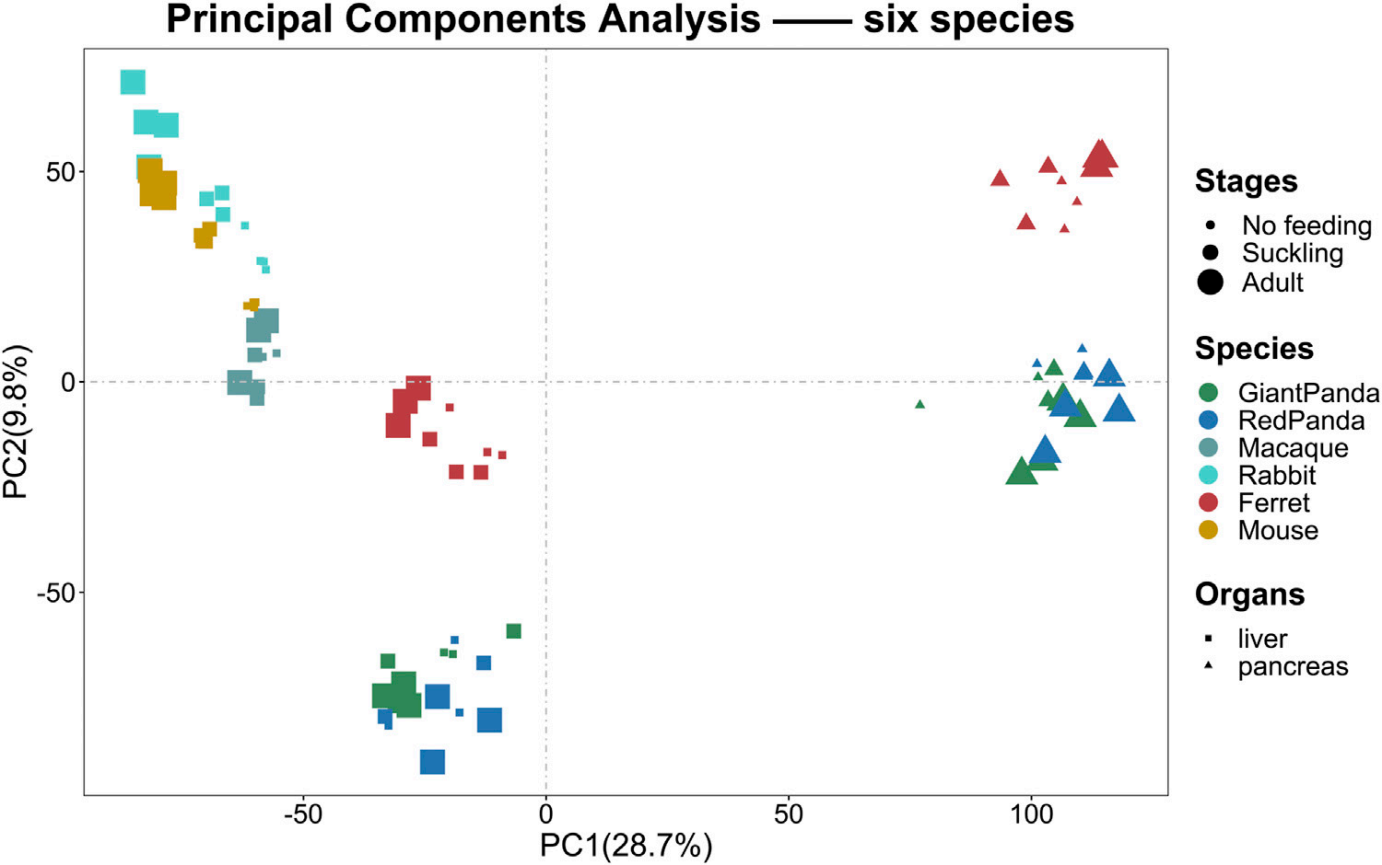
PCA analyses of the expression for liver or pancreas samples among six species. PCA of the log-transformed normalized CPM expression levels of all liver or pancreas samples. Species are represented by different colors. Stages are represented by the size of the point. Organs are represented by different shapes.

**FIGURE 2:**
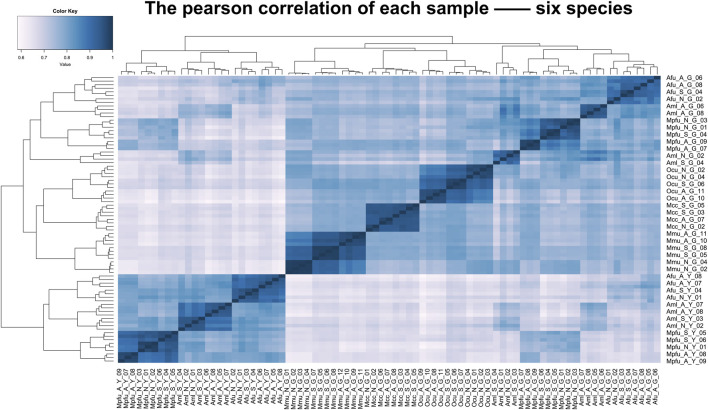
Clustering analyses of the expression for liver and pancreas samples among six species. Clustering of liver and pancreas samples among six species based on log-transformed normalized CPM expression values. Distance between samples was measured by Spearman’s rank correlation coefficient.

**FIGURE 3:**
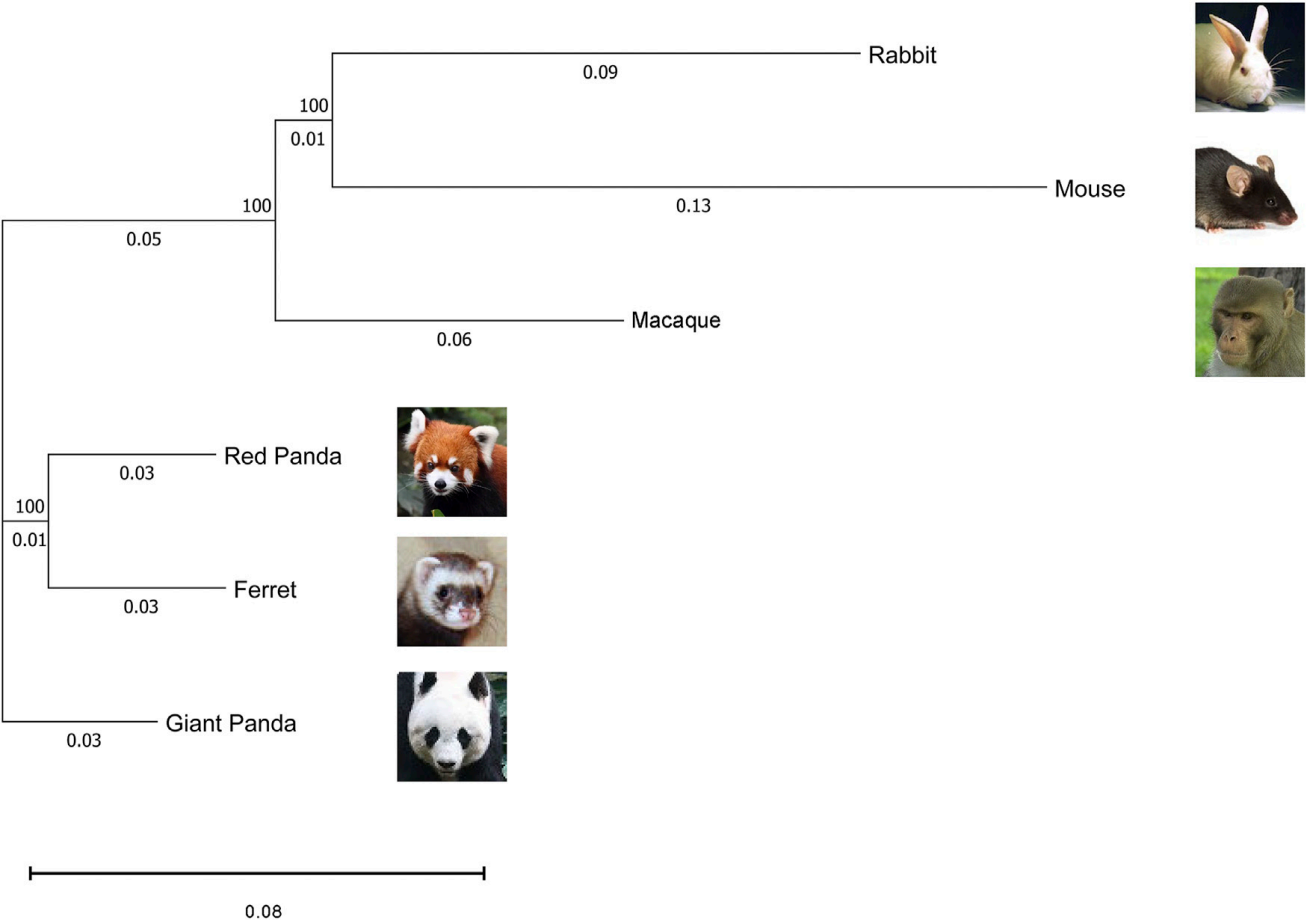
Maximum likelihood phylogeny of the six species in this study based on 1:1 single-copy orthologues coding sequences. The numbers in the nodes represent the bootstrap values. The scale bar indicates the number of substitutions per site.

To assess the grouping relationships and clustering patterns among different species, we performed principal component analysis (PCA) and spearman correlation distance clustering analysis based on the homologous gene expression matrices of six species (Figures 1, 2). The results of PCA found that liver samples and pancreas samples could be completely separated among different species, indicating that organ functions evolved relatively conservatively during the evolution of mammals. Samples from the suckling group of each species were almost clustered with samples from their adult and no feeding groups, indicating that mammals still have a large species specificity in the gene expression patterns of important digestive organs despite the fact that they all feed on breast milk at a young age. While in different organs, red pandas were clustered together with giant pandas, which are also bamboo eaters but are more distantly related and could not be separated, although red pandas and ferrets have relatively close affinity (Figure 3). In addition, the omnivorous mice and the herbivorous rabbits and macaques showed closer expression patterns than other dietary species. The carnivorous ferrets, on the other hand, differed more from the rest of the non-carnivorous species in their expression patterns. Similar to the results of PCA, the results of spearman’s correlation distance clustering were also divided into two main stems and several branches according to tissue and species in turn. In the pancreas, red pandas were clustered first with giant pandas, which were similar in diet but relatively distant in affinity, and then with ferrets, which were similar in affinity but different in diet. Similarly, samples of red pandas in the liver first clustered with samples from the adult giant panda group, then clustered with ferret samples, and finally clustered with samples from the suckling and no feeding groups of giant pandas, suggesting that the expression pattern of juvenile giant panda livers is relatively specific and undergoes a large degree of change from juvenile to adult. In addition, except for the livers of red pandas, mice, and macaques, the suckling group samples of liver or pancreas from all species clustered with the no feeding group samples before clustering together with the adult group samples. In contrast, in the livers of giant pandas, mice, and macaques, the suckling group samples clustered with the adult group samples before clustering with the no feeding group samples.

### 3.2 Identification of differentially expressed genes (DEGs)

**FIGURE 4:**
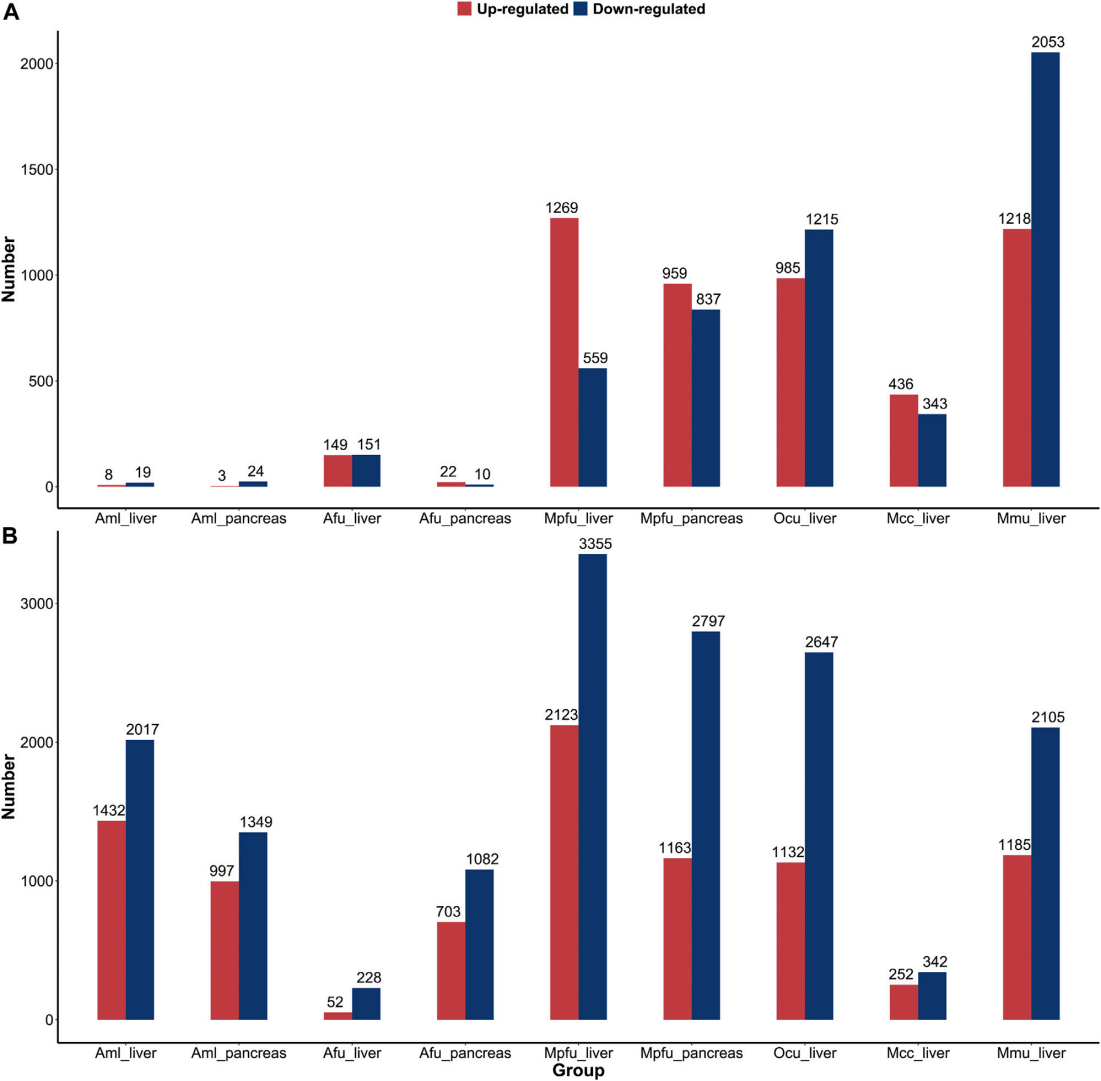
Distribution of differentially expressed genes of six species’ liver or pancreas. **(A)** The number of upregulated and downregulated DEGs in the suckling group compared to the no feeding group among six species. **(B)** The number of upregulated and downregulated DEGs in the adult group compared to the suckling group among six species. Abbreviations: Aml: giant panda, Afu: red panda, Mpfu: ferret, Ocu: rabbit, Mcc: macaque, Mmu: mouse.

We performed a comparative analysis of gene expression during the suckling stage with no feeding stage and adult stage in six species, respectively, in order to characterize the postnatal changes in gene expression of important digestive and metabolic organs in different feeding mammals (Figure 4). In comparison to the suckling and no feeding groups, the number of DEGs in the liver and pancreas of giant pandas and pancreas of red pandas was very low, much lower than the number of DEGs in other dietary species. This indicates that gene expression was fully open at birth in the liver and pancreas of giant pandas and the pancreas of red pandas, while the corresponding organs of other species were significantly altered by food (milk) stimulation. Whereas, in the comparison between the suckling group and the no feeding group of liver in red pandas, although the number of DEGs was similarly lower than in other species except giant pandas, it was significantly higher than in the pancreas. This indicates that the liver of red pandas is born with relatively low functional development compared to the pancreas. In the comparison between the adult and suckling groups, the number of DEGs was relatively high in the liver or pancreas of all species except the livers of macaques and red pandas. Suggesting that the livers of red pandas and macaques mature rapidly in function during suckling with little difference in expression from adult period. Surprisingly, the number of DEGs in carnivore ferrets was quite large in the liver and pancreas of adult groups compared to the suckling groups, despite the fact that they fed on a high-fat, high-protein, low-carbohydrate diet during both suckling and adult stages. This suggests that the liver and pancreas of ferrets undergo a greater functional adjustment in adulthood.

### 3.3 Functional enrichment analysis of DEGs

We performed GO and KEGG enrichment analyses of DEGs from the liver or pancreas of six species, respectively, to further understand the changes of function in the liver and pancreas from juvenile to adult.

The results of KEGG enrichment of DEGs in the liver and pancreas of the bamboo-eating giant pandas are shown in Supplementary Figure S1, and the results of GO enrichment are shown in Supplementary Tables S2–S9. We found that in the comparison of liver samples of giant pandas between different stages, a large number of immune-related entries were significantly enriched in the upregulated genes in the adult group compared to the suckling group and the upregulated genes in the suckling group compared to the no feeding group. Although the liver is not a lymphoid organ, it contains many cells involved in immune functions and assumes a certain function in body immunity (Kubes and Jenne, 2018). In the comparison between the suckling group and the no feeding group, there were relatively few digestive and metabolic related entries significantly enriched in giant pandas. Among them, the downregulated genes in the liver samples of giant pandas in the suckling group compared to the no feeding group were significantly enriched for entries such as cholesterol biosynthetic process (GO:0006695), and the upregulated genes in the pancreas samples of the suckling group compared to the no feeding group were significantly enriched for entries such as carbohydrate biosynthetic process (GO:0016051). In the adult group compared to the suckling group, a large number of entries related to digestion and metabolism were significantly enriched in the giant pandas. Compared to the liver samples in the suckling group, the liver samples in the adult group of giant pandas were enriched for entries related to carbohydrate metabolism such as starch and sucrose metabolism (PATH:ko00500), carbohydrate digestion and absorption (PATH:ko04973), and lipid metabolism such as arachidonic acid metabolism (PATH:ko00590) and linoleic acid metabolism (PATH:ko00591). The enhancement of carbohydrate metabolism in the liver of giant pandas after weaning helps them to fully utilize the available carbohydrates in bamboo to meet the energy demand of the body. The upregulated genes in the liver samples of the adult group were also significantly enriched for the P450 related pathways such as metabolism of xenobiotics by cytochrome P450 (PATH:ko00980) and drug metabolism—cytochrome P450 (PATH:ko00982), which help metabolize plant secondary metabolites (PSMs) in bamboo and mitigate the negative effects of high intake of bamboo (Jandacek, 2017; Burns et al., 2018; Henderson et al., 2018). In addition, compared to the suckling group, the upregulated genes in the pancreas samples of the adult group were significantly enriched for arginine biosynthesis (PATH:ko00220) pathway, and the downregulated genes in the liver samples of the adult group were significantly enriched for lysine degradation (PATH:ko00310) pathway. This is beneficial for meeting the basic requirements of arginine and lysine in the low amino acid diet of giant pandas.

The results of KEGG enrichment of DEGs in the liver and pancreas of the bamboo-eating red pandas are shown in Supplementary Figure S2, and the results of GO enrichment are shown in Supplementary Tables S10–S17. We found that, similar to the giant pandas, a large number of immune-related entries were significantly enriched in the upregulated genes in the red pandas liver samples of the adult group compared to the suckling group and in the suckling group compared to the no feeding group. In the comparison between the suckling and no feeding groups, red pandas were also significantly enriched for few digestive metabolism related entries. Among them, upregulated genes were significantly enriched for glycolysis/gluconeogenesis (PATH:ko00010), pyruvate metabolism (PATH:ko00620) and other carbohydrate related pathways in the liver samples of suckling group compared to the no feeding group. In comparison between the adult and suckling group samples, the downregulated genes were significantly enriched for lipid metabolism pathways such as cholesterol metabolism (PATH:ko04979) and acylglycerol metabolic process (GO:0006639) in adult pancreas samples. The genes upregulated in pancreas samples of the adult group were significantly enriched for regulation of glucose metabolic process (GO:0010906), regulation of gluconeogenesis (GO:0006111), monosaccharide biosynthetic process (GO:0046364) and lipid synthesis-related entries such as lipoprotein biosynthetic process (GO:0042158). In addition, similar to giant pandas, the arginine biosynthesis (PATH:ko00220) pathway and glutamine family amino acid biosynthetic process (GO:0009084) were significantly enriched in upregulated genes in pancreas samples from the adult group of red pandas compared to the suckling group, as an adaptive response to a low amino acid bamboo diet.

The results of KEGG enrichment of DEGs in the liver and pancreas of carnivorous ferrets are shown in Supplementary Figure S3, and the results of GO enrichment are shown in Supplementary Tables S18–S25. The immune-related entries were significantly enriched in higher-expressed genes in the liver or pancreas samples from the ferrets suckling group compared to the no feeding group, and also significantly enriched in higher-expressed genes in pancreas samples from the adult group compared to the suckling group. In addition to those immune-related entries, the liver and pancreas samples of ferrets were also significantly enriched with a large number of digestive and metabolic entries in the comparison between different growth and developmental stages. Among them, the downregulated genes in the liver samples from the suckling group were mainly enriched for lipid synthesis-related pathways such as biosynthesis of unsaturated fatty acids (PATH:ko01040), lipid biosynthetic process (GO:0008610) and cholesterol biosynthetic process (GO:0006695) than those from the no feeding group. The upregulated genes in the liver samples of the suckling group were mainly enriched for protein digestion and absorption (PATH:ko04974) and cholesterol metabolism (PATH:ko04979) than the liver samples of the no feeding group. In addition, the upregulated genes in the pancreas samples of the suckling group were significantly enriched for a large number of entries related to lipid catabolism, such as fatty acid beta-oxidation (GO:0006635), fatty acid degradation (PATH:ko00071), and triglyceride lipase activity (GO:0004806), than those in the pancreas samples of the no feeding group. During the suckling stage, the highly expressed of a large number of digestive metabolism related genes in ferrets’ liver and pancreas facilitates their full utilization of nutrients in breast milk. Interestingly, although ferrets were fed a high-fat, high-protein diet both before and after weaning, the number of DEGs identified and digestive metabolism related entries enriched in the liver and pancreas was very high between the adult and suckling groups. For example, the lower expressed genes in the liver samples of the adult group of ferrets were significantly enriched for carbohydrate digestion and absorption (PATH:ko04973), negative regulation of peptidase activity (GO:0010466), and serine-type endopeptidase inhibitor activity (GO:0004867) compared to the suckling group. The lower expressed genes in pancreas samples from the adult group were mainly enriched for lipid biosynthesis proteins (PATH:ko01004), biosynthesis of unsaturated fatty acids (PATH:ko01040) and other lipid synthesis-related entries. The highly expressed genes in the liver samples from the adult group of ferrets were also significantly enriched for a large number of digestive metabolism related entries, carbohydrate metabolism pathways such as citrate cycle (TCA cycle) (PATH:ko00020); lipid metabolism pathways such as fatty acid degradation (PATH:ko00071), fatty acid beta-oxidation (GO:0006635); alanine, aspartate and glutamate metabolism (PATH:ko00250), glycine, serine and threonine metabolism (PATH:ko00260), and other amino acid metabolism related pathways. The genes highly expressed in the pancreas samples of the adult group of ferrets were significantly enriched for protein digestion and absorption (PATH:ko04974), serine-type endopeptidase activity (GO:0004252) and other protein and amino acid metabolism related pathways.

The results of KEGG enrichment of DEGs in the liver of herbivorous rabbits are shown in Supplementary Figure S4, and the results of GO enrichment are shown in Supplementary Tables S26–S29. The enrichment results showed that the higher expressed genes in the liver samples of the rabbit suckling group compared to the no feeding group were significantly enriched for fatty acid degradation (PATH:ko00071), cholesterol metabolism (PATH:ko04979), serine-type endopeptidase activity (GO:0004252), tyrosine metabolism (PATH:ko00350) and other lipid utilization and amino acid metabolism related entries. The highly expressed genes related to lipid and amino acid metabolism in the liver of suckling rabbits help them to fully utilize the lipids and proteins rich in breast milk. In addition, the upregulated genes in the liver samples of the adult group of rabbits were mainly enriched for amino acid metabolism related entries such as arginine biosynthesis (PATH:ko00220) and tryptophan metabolism (PATH:ko00380) compared to the suckling group.

The results of KEGG enrichment of DEGs in the liver of herbivorous macaques are shown in Supplementary Figure S5, and the results of GO enrichment are shown in Supplementary Tables S30–S33. The enrichment results showed that the higher expressed genes in the liver samples of macaques in the suckling group compared to the no feeding group were significantly enriched for cholesterol metabolic process (GO:0008203), steroid hormone biosynthesis (PATH:ko00140), tyrosine metabolism (PATH:ko00350) and other entries related to lipid utilization and amino acid metabolism. The highly expressed genes related to lipid metabolism and amino acid metabolism in the livers of macaques during suckling stage contribute to the full utilization of lipids and proteins rich in breast milk. Compared to the suckling group, the upregulated genes in the liver samples of adult group were mainly enriched for pathways such as steroid hormone biosynthesis (PATH:ko00140), retinol metabolism (PATH:ko00830), and metabolism of xenobiotics by cytochrome P450 (PATH:ko00980), and the downregulated genes were mainly enriched for negative regulation of gluconeogenesis (GO:0045721) and other entries.

The results of KEGG enrichment of DEGs in the liver of omnivorous mice are shown in Supplementary Figure S6, and the results of GO enrichment are shown in Supplementary Tables S34–S37. The upregulated genes in the liver samples of the mice in the suckling group were significantly enriched for immune-related entries compared to the liver samples in the no feeding group. In addition, a large number of digestive metabolism related entries were also significantly enriched in the comparison between the suckling group and no feeding group. For example, the genes that were lower expressed in the suckling group than in the no feeding group were significantly enriched for cholesterol biosynthetic process (GO:0006695), steroid biosynthetic process (GO:0006694), and endopeptidase inhibitor activity (GO:0004866). The genes that were highly expressed in the suckling group compared to the no feeding group were significantly enriched for pathways such as pyruvate metabolism (PATH:ko00620), fatty acid degradation (PATH:ko00071), and glycine, serine and threonine metabolism (PATH:ko00260). Similarly, the genes significantly upregulated in the liver samples of the adult group of mice were also significantly enriched for a large number of digestive metabolism related entries when compared to the suckling group. For example, glycolysis/gluconeogenesis (PATH:ko00010), pyruvate metabolism (PATH:ko00620), glycogen biosynthetic process (GO:0005978), fatty acid degradation (PATH:ko00071), lipid catabolic process (GO:0016042), triglyceride lipase activity (GO:0004806), biosynthesis of unsaturated fatty acids (PATH:ko01040), alanine, aspartate and glutamate metabolism (PATH:ko00250), glycine, serine and threonine metabolism (PATH:ko00260) and other entries. The overall upregulation of genes related to carbohydrate, lipid, protein and amino acid metabolism in the liver of adult mice contributes to their full utilization of various nutrients in food.

### 3.4 Identification of key genes for digestion and metabolism

**FIGURE 5:**
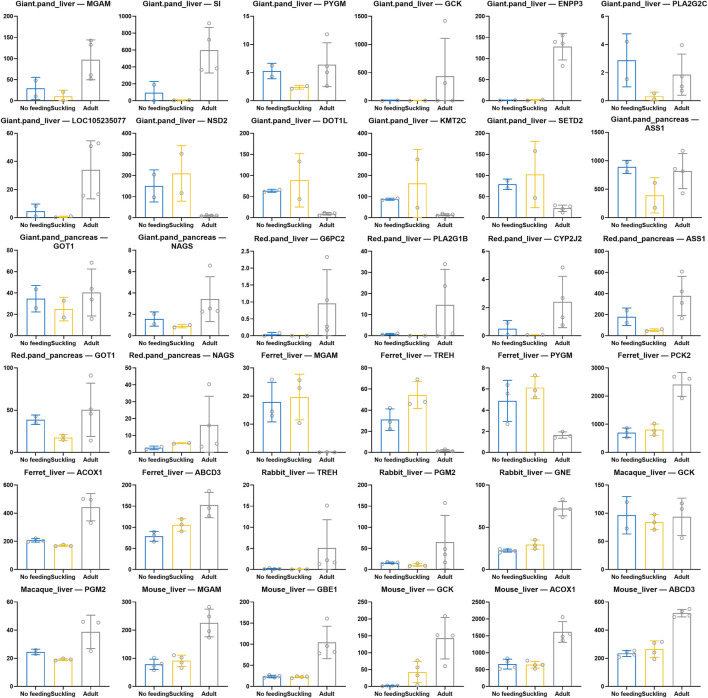
Scatter bar chart of expression of key genes related to digestive and metabolism among six species. X-axis indicates different comparison groups and Y-axis indicates CPM normalized expression.

Based on the functional enrichment results, we extracted and identified key genes for digestion and metabolism in each species (Figure 5). We found convergent patterns of expression of key genes for starch and sucrose metabolism in the livers of giant and red pandas, for example, MGAM, SI, PYGM, GCK, and ENPP3 were significantly highly expressed in the livers of adult giant pandas, and G6PC2 was significantly highly expressed in the livers of adult red pandas. Similarly, key genes for starch and sucrose metabolism were also significantly highly expressed in adult macaque, rabbit and mouse livers. For example, macaque: GCK, PGM2; rabbit: TREH, PGM2, GNE; mouse: MGAM, GBE1, GCK. In contrast, the key genes for starch and sucrose metabolism, MGAM, TREH, and PYGM, were significantly less expressed in the livers of adult ferrets. Among the genes related to lipid metabolism, we found that the key genes for fatty acid β-oxidation, ACOX1 and ABCD3 showed convergent high expression patterns in adult ferret liver and mouse liver. In the adult group of giant and red pandas, there were convergently high expression patterns of key genes for unsaturated fatty acid metabolism in linoleic acid and arachidonic acid, which are rich in plants. Such as, giant pandas: PLA2G2C, LOC105235077; red pandas: PLA2G1B, CYP2J2. Among amino acid and protein metabolism related genes, we found that key genes for arginine biosynthesis (ASS1, GOT1, NAGS) had a convergent high expression pattern in the pancreas of adult giant and red pandas. The lysine degradation key genes NSD2, DOT1L, KMT2C, and SETD2 were significantly less expressed in the liver of adult giant pandas.

To further validate the reliability of the results, we analyzed the expression of fatty acid β-oxidation related DEGs (ACADM, ACOX1, HADH) in the livers of red pandas and ferrets, the expression of sphingolipid metabolism related DEG SPHK2 in the livers of red pandas and the expression of carbohydrate metabolism related DEG FBP1 in ferrets. In the above analysis, we found that the expression of ACADM, ACOX1 and HADH in liver samples from the adult group of ferrets was significantly higher than that in the suckling group, but there was no significant difference between the expression in liver samples from the adult group and the suckling group of red pandas. In addition, the expression of SPHK2 in liver samples from the adult group of red pandas and the expression of FBP1 in liver samples from the adult group of ferrets were significantly higher than those from the suckling group. As shown in Supplementary Figure S7, the results of RT-qPCR revealed similar expression tendency. Despite some quantitative differences at the expression level, the result of RT-qPCR indicated that the expression patterns of nutrition metabolism-related genes were reliable.

## 4 Discussion

### 4.1 Adaptive relationship between gene expression feature in non-feeding juveniles and pandas’ bamboo-eating diets

The results of this study showed that the number of DEGs between suckling stage and no feeding stage in the liver and pancreas samples of giant and red pandas was low and much lower than that of other dietary species, which seems to contradict their rapid growth and development after birth. Giant pandas have a very short post-implantation gestation period, which produces very young neonates (Zhang et al., 2009; Li and Smith, 2020) and more than 99% of body growth occurs postnatally (Grand, 1992), with a 900:1 ratio to maternal weight (Huang et al., 2018). Similarly, more than 89% of body growth in red pandas occurs postnatally (Grand, 1992). Among eutherian mammals, bears produce the most altricial neonates with the smallest juvenile-maternal body weight ratio, which is thought to address the energy conservation needs of mothers with reduced metabolism during hibernation (Ewer, 1973; Ramsay and Dunbrack, 1986). However, some bears, like giant pandas and sloth bears, do not hibernate (Gerstner et al., 2016; Bi et al., 2021). Meanwhile, among ursidae species, giant pandas represent one of the most extreme examples, producing the most immature neonates (Zhang et al., 2009). The development of the giant panda embryo after implantation is extremely short, being detectable only 15–20 days before the mother delivers (Zhang et al., 2009), and the gestation period is the shortest among all ursidae species. This may be an adaptive response of giant panda reproduction to the bamboo diet, which is a low-nutrient, low-energy food with a relative lack of protein and lipids (Li et al., 2000). During gestation, the fetus is unable to catabolize and metabolize maternal free fatty acids, and the mother is forced to use her own proteins to replace the fetal gluconeogenesis pathway, which is a more energy-consuming process compared to the supply of breast milk (Ramsay and Dunbrack, 1986; Ewer, 1998). Fetal development within the mother may be limited when maternal nutrients are relatively deficient, and cubs produce rapidly in order to obtain nutritional supplies from breast milk (Li and Smith, 2020). Whereas the body weight growth rate and milk intake of giant pandas increase rapidly within 10 days of birth (Huang et al., 2018), the protein content in giant pandas’ breast milk is significantly higher in the early period (3–6 days) than in the later period (7–23 days), facilitating the cubs’ rapid growth (Huang et al., 2018). In this study, giant panda suckling cubs were 4 and 6 days old, which implies a high nutritional requirement during this period. The low number of DEGs between no feeding and suckling groups of liver and pancreas in giant pandas may be attributed to the fact that genes related to nutrient metabolism were already open at birth to meet the needs of rapid postnatal growth and development in giant pandas. Probably for the same reason, the DEGs in no feeding and suckling groups have similar trends to those in giant pandas. Regardless, although ferrets are phylogenetically more closely related to red pandas, the DEGs between no feeding and suckling groups of ferrets did not exhibit similar characteristics to those of red pandas (Figure 4A). Therefore, the expression patterns of genes in no feeding and suckling groups of giant and red pandas may be an indication of their reproductive strategy in response to a low-nutrient bamboo diet, as a trade-off for energy expenditure. In addition, the number of DEGs was similar between the liver and pancreas of giant pandas, while the number of DEGs was higher in the liver than in the pancreas of red pandas in the comparison of the suckling and no feeding groups, despite the fact that the individuals compared were identical. This may be due to heterochrony in the development of the organs (Cardoso-Moreira et al., 2019).

### 4.2 Expression change patterns of genes related to digestion and metabolism from suckling to adult stage in different dietary mammals

During the juvenile period, different dietary mammals all feed on breast milk, which is rich in proteins, lipids, and certain amounts of carbohydrates such as lactose and galactose (Girard et al., 1992; Nakamura et al., 2003; Zhang et al., 2016). However, in adult period, the diets of mammals are much more diverse and can be broadly classified as carnivorous, herbivorous, and omnivorous. Although giant pandas and red pandas are carnivores, giant and red pandas have undergone a dramatic change in diet during evolution, from a high-fat, high-protein meat diet to a low-fat, low-protein bamboo diet (Zhang et al., 2016; Jin et al., 2020; Glatston, 2021). Compared to the lipid-rich meat and high soluble carbohydrate content of the stems and leaves of young tender plants, the composition of bamboo is predominantly cellulose, containing less than 2% soluble carbohydrates, 3.8% protein, and 0.3% lipids (Girard et al., 1992). Does this predict that giant and red pandas may have unique gene expression change patterns that differ from both carnivores and general herbivores.

We found that the expression of key genes for starch and sucrose metabolism was significantly higher in the liver of the adult group of bamboo-eating giant pandas (*MGAM*, *SI*, *PYGM*, *GCK*, *ENPP3*, etc.) and red pandas (*G6PC2*, etc.) than in the suckling group. *MGAM* and *SI* play important roles in the final digestion of starch (Calvo et al., 2000; Ghadyale et al., 2012; Nalawade et al., 2019), *PYGM* and *G6PC2* are key glycogenolytic enzymes (Al-Daghri et al., 2017; Migocka-Patrzałek et al., 2020). Similarly, key genes involved in starch and sucrose metabolism are found highly expressed in the livers of adult groups of herbivore macaques (*GCK*, *PGM2*, etc.) and rabbits (*TREH*, *PGM2*, *GNE*, etc.). *GCK* encodes glucokinase, and *PGM2* encodes phosphoglucose metatase, both are key enzymes for glucose catabolism, and *TREH* encodes a key enzyme for hydrolysis of alginose in plants (Oesterreicher et al., 2001; Nardelli et al., 2019). In omnivore mice, the expression of key genes for starch and sucrose metabolism (*MGAM*, *GBE1*, and *GCK*) was also significantly higher in the liver of adult group than in suckling group. However, unlike the mammals mentioned above, the expression of key genes for starch and sucrose metabolism (*MGAM*, *TREH*, *PYGM*, etc.) in the livers of carnivore ferrets was significantly lower in the adult group compared to the suckling group. Soluble carbohydrates such as starch and sucrose are important energy substances in plant-based foods. In contrast, in meat-based foods, the main energy substrate is glucose stored as glycogen (Migocka-Patrzałek et al., 2020), which does not contain carbohydrates such as starch and sucrose, which are unique in plant-based foods. This suggests that the bamboo-eating giant and red pandas possess similar starch and sucrose hydrolysis abilities as herbivores like rabbits and macaques, and omnivores like mice, which have plant components in their food. This result is also consistent with the higher copy number of the starch hydrolase gene in giant pandas than in carnivores (Zhang et al., 2018). Diet has an adaptive shaping effect on genes (Mathieson, 2020; Rees et al., 2020). Therefore, the high expression of key genes for starch and sucrose metabolism in giant and red pandas after weaning is an adaptive response to the bamboo diet, which facilitates them to fully utilise the available carbohydrates in bamboo and alleviate the energy shortage. Besides, key genes involved in carbohydrate digestion and absorption were also significantly highly expressed in adult groups of giant and red pandas, rabbits, macaques, and mice, which were also beneficial to enhance their utilisation of carbohydrates in food. In the liver of adult ferret, key genes for glycolysis/glycogenesis and citric acid cycle (*FBP1*, *ADH4*, *PCK2*, etc.) were significantly highly expressed. This contributes to the catabolism and utilisation of glycogenic substances in the meat diet of adult ferrets.

Fatty acid β-oxidation is the primary pathway of fatty acid oxidative catabolism in the body, supplying a significant amount of energy to the organism. We found that key genes for fatty acid β-oxidation (*ACOX1*, *ABCD3*, *ACADM*, *HADH*, etc.) were significantly highly expressed in the liver of adult ferrets. Mice are also feed on lipid-rich foods after weaning, such as plant seeds and meat foods. The key genes for fatty acid β-oxidation (*ACOX1*, *ACOX3*, *ABCD3*, etc.) were significantly highly expressed in the adult group of mice livers, as they were in ferrets. The genes mentioned above are key enzymes in the fatty acid oxidation step, catalysing the oxidation of fatty acids and playing an important role in fatty acid metabolism for energy supply (Chung et al., 2020; Ma et al., 2021; Ranea-Robles et al., 2021). The high expression of key genes for fatty acid β-oxidation in ferrets and mice during adulthood contributes to the adequate metabolism and utilization of lipids abundant in food. Interestingly, the expression of key genes for fatty acid β-oxidation in the livers of giant and red pandas did not differ significantly between the no feeding, suckling, and adult groups. This implies that fatty acids may not be the main source of energy during suckling in giant and red pandas and that the main source of energy during suckling may be carbohydrates such as lactose and galactose, which are abundant in breast milk (Ballard and Oliver, 1963; Walker and Snell, 1973; Girard et al., 1992; Nakamura et al., 2003; Zhang et al., 2016). Fatty acids in breast milk during suckling period may be mainly used for other important physiological functions, for example, neuronic acid is essential for brain development and function (Nilsson et al., 2018), and docosahexaenoic and arachidonic acids are essential for visual, motor, and cognitive development (Birch et al., 1992; Fang et al., 2005; Innis, 2008; Qawasmi et al., 2013; Tam et al., 2016; Wang et al., 2016). In addition, preterm infants have a high requirement for arachidonic and docosahexaenoic acids because they have not stored enough fat during fetal life (Harris et al., 2019). Compared to the suckling group, key genes for fatty acid β-oxidation in the liver of adult giant and red pandas were also not significantly highly expressed. After weaning, the lipid contents of the bamboo food are very low (Girard et al., 1992; Zhang et al., 2016; Jin et al., 2020; Glatston, 2021). As a result, the expression pattern of fatty acid β-oxidation related genes in adults is consistent with their lipid-deficient diet. Studies have shown that the addition of relatively high amounts of animal-derived foods to the giant panda feed can lead to metabolic disorders, resulting in lethargy, frequent mucus excretion (4–10 times/month), lack of appetite, and reduced activity (Knight et al., 1982). We speculate that during the evolution of giant and red pandas from ancestral carnivorous to bamboo-eating species, fatty acid oxidation related pathways may have undergone negative selection and oxidative catabolic capacity gradually weakened, ultimately leading to their inability to adapt to high-fat foods. Similarly, key genes for fatty acid β-oxidation in rabbits and macaques livers were not significantly different between adult groups and suckling groups. This is their response to low-fat plant foods. Although fatty acid oxidative catabolism was relatively weak in the livers of adult giant and red pandas, rabbits, and macaques, significant high expression of key genes for lipid metabolism (*PLA2G1B*, *PLA2G2C*, *CYP2J2*, *UGT2B16*, *HSD11B1*, etc.) such as arachidonic acid metabolism and linoleic acid metabolism were also present. Plant foods have much higher levels of linoleic acid (LA) and α-linolenic acid (ALA) than animal meat, and arachidonic acid (AA) can be obtained through LA conversion. The significantly high expression of the above genes facilitates the full utilisation of unsaturated fatty acids rich in the foods of herbivorous animals (Abedi and Sahari, 2014; Taha et al., 2017). Aside from fatty acid oxidation, adult ferrets and mice had high expression of key genes for lipid metabolism such as fatty acid degradation (*EHHADH*, *ALDH9A1*, *ALDH2*, etc.), which allowed them to fully exploit the lipids rich in meat food.

In giant and red pandas, key genes for arginine biosynthesis (*ASS1*, *GOT1*, *NAGS*, etc.) were significantly highly expressed in pancreas samples from the adult group. The content of arginine in bamboo is very low (Hu et al., 2017; U.S. Department of Agriculture, 2019a) and the high expression of key genes for arginine biosynthesis after weaning helps to meet the basic requirement of arginine in giant and red pandas under low arginine diet. Furthermore, in giant panda livers, the expression of key genes for lysine degradation (*NSD2*, *DOT1L*, *KMT2C*, *SETD2*, etc.) was significantly lower in adult group than in suckling group, and the above genes were able to regulate the methylation levels of lysine residues during lysine degradation. Like arginine, the level of lysine in bamboo is very low (Hu et al., 2017; U.S. Department of Agriculture, 2019a). Therefore, the low expression of lysine degradation genes in adult giant pandas may be beneficial in meeting the body’s lysine requirement and maintaining the homeostasis of lysine in body under a low lysine diet (Cleveland et al., 2008). The improvement of arginine synthesis capacity in adult giant and red pandas and the downregulation of lysine degradation capacity in adult giant pandas are both features of the adaptive response under a low amino acid bamboo diet. In rabbits, macaques, ferrets and mice, the livers of their adult groups exhibit a significant pattern of high expression of a large number of key genes for amino acid metabolism because of the abundance of amino acids in their adult diets.

Interestingly, despite the relatively similar nutritional composition of the food, the number of DEGs and digestive metabolism related entries enriched in the liver and pancreas of ferrets were very high in adult group compared to suckling group. Ferrets feed on breast milk before weaning and on meat from rodents, rabbits, and poultry after weaning (Harrer and Schmidt, 1986; Ragg et al., 2000; Davis and Williams, 2017). Both milk and meat are high-fat, high-protein foods and are more similar in amino acid and fatty acid composition (Schoknecht et al., 1985; Jenness, 1974; Ahamad et al., 2017; U.S. Department of Agriculture, 2019b; U.S. Department of Agriculture, 2019c; Lu et al., 2019). We found that in ferret liver, a large number of key genes involved in lipid metabolism as well as amino acid and protein metabolism showed a significantly high expression pattern, such as those involved in fatty acid biosynthesis, fatty acid degradation, tyrosine metabolism, and phenylalanine metabolism. Similarly, in the ferret pancreas, key genes for amino acid and protein metabolism and key genes for some lipid metabolism also showed significantly high expression patterns. Ferrets and other mustela species have a high basal metabolic rate (Iversen, 1972; Boyce et al., 2001; Aubry et al., 2012). In addition, due to the low digestibility caused by a very short gut, ferrets need to eat and consume large amounts of high-fat, high-protein meat foods constantly on a daily basis, and the excess intake does not cause serious adverse effects (Bell, 1999; Boyce et al., 2001). As a result, we hypothesise that in adult ferrets, the overall upregulation of key genes for substance metabolism, as well as the high number and amount of daily intake, are intended to offset the negative effects of low digestibility and a high basal metabolic rate.

## 5 Conclusion

In summary, we find that the expression patterns of digestion and metabolism related genes in the herbivorous, bamboo-eating, carnivorous, and omnivorous animals in this study all exhibit characteristics adapted to their food habits. In addition to sharing similar expression changes with the usual herbivores rabbits and macaques, giant and red pandas exhibit expression characteristics associated with specific nutritional limitations of bamboo. The key genes for starch hydrolysis and sucrose metabolism, lipid metabolism, and protein metabolism in the omnivore mouse showed a significant high expression pattern in adulthood. In contrast to what is generally expected, there were a large number of DEGs between the adult group and the suckling group in carnivore ferrets, with significantly high expression of genes related to lipid, amino acid, and protein metabolism, which may be associated with high metabolic levels in adult carnivores. Our study systematically elucidates the pattern of changes in gene expression related to digestion and metabolism from juvenile to adult in different dietary mammals.

## Data Availability

The datasets presented in this study can be found in online repositories. The names of the repository/repositories and accession number(s) can be found in the article/Supplementary Material S1.
