# Supplementary material for: Comparative study of the digestion and metabolism related genes’ expression changes during the postnatal food change in different dietary mammals
Source: Front Genet. 2023 Jul 4;14:1198977. doi: 10.3389/fgene.2023.1198977 (PMC10352678; doi:10.3389/fgene.2023.1198977)
Supplement: Supplementary file 1 [file DataSheet1.zip › Supplementary Figures/Supplementary Figures.docx]

Supplementary Material

Comparative study of the digestion and metabolism related genes' expression changes during the postnatal food change in different dietary mammals

**Zhuma Yizhen^1†^, Lei Chen^2†^, Xiaodie Jie^2^, Fujun Shen^3^, Liang Zhang^3^, Yusen Hou^4^,** **Lu Li^2^,** **Guoqiang Yan^2^, Xiuyue Zhang^2^, Zhisong Yang^1*^**

^1^Sichuan Academy of Giant Panda, 1375 Panda Road, Northern Suburb, Chengdu 610081, China

^2^Sichuan Key Laboratory of Conservation Biology on Endangered Wildlife, College of Life Sciences, Sichuan University, Chengdu 610064, China

^3^Sichuan Key Laboratory for Conservation Biology of Endangered Wildlife, Chengdu Research Base of Giant Panda Breeding, 1375 Panda Road, Northern Suburb, Chengdu 610081, China

^4^Pharmacy College, Chengdu University of Traditional Chinese Medicine, Sichuan, China

***Correspondence:**

Zhisong Yang,

yangzhisong@126.com

# Supplementary Figures

## Supplementary Figures

**
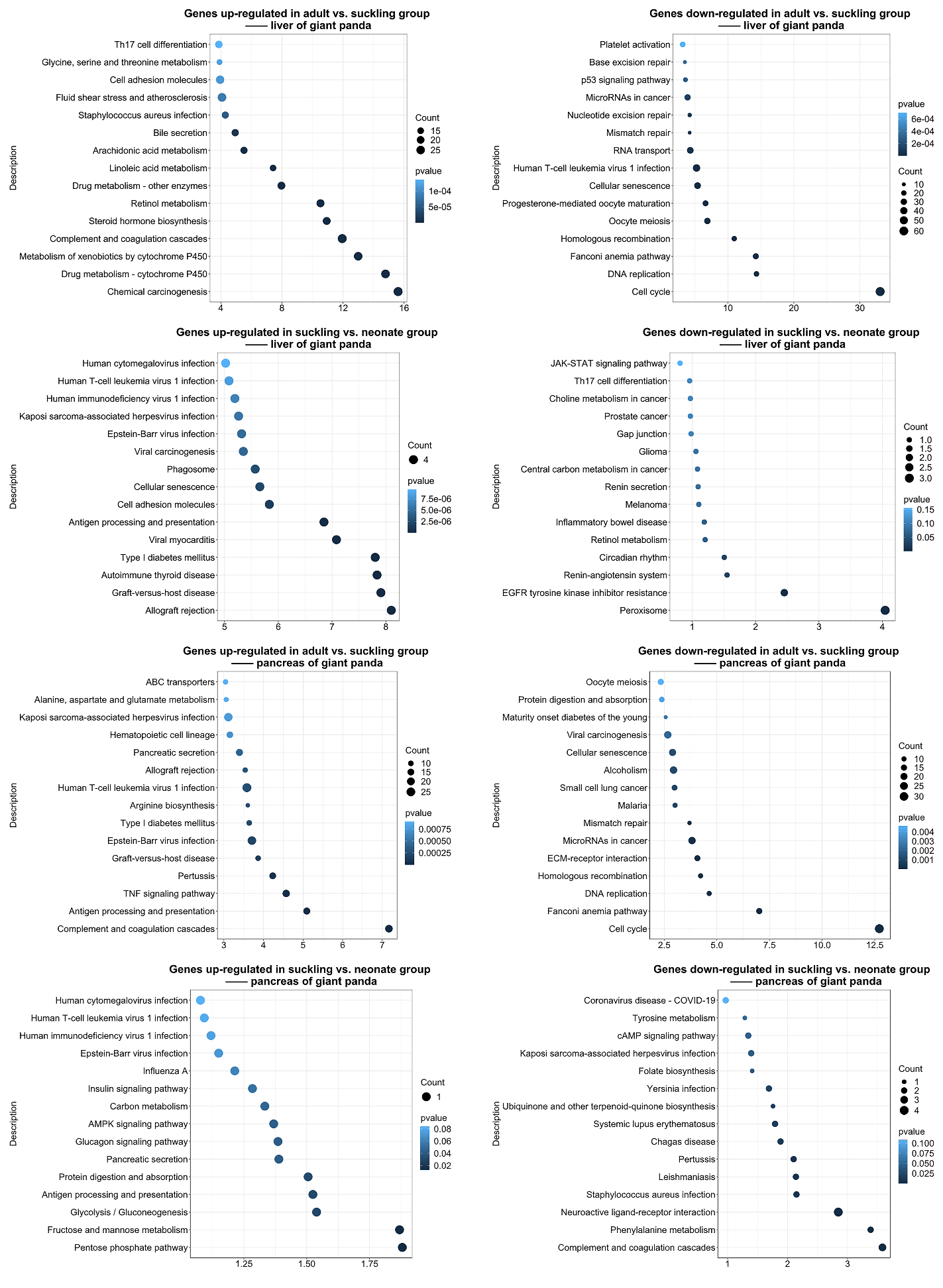
**

**Supplementary Figure 1.** KEGG enrichment results of DEGs in the liver or pancreas of giant pandas. X-axis indicates the -log10(P) of KEGG enriched pathways, Y-axis indicates the name of the pathway. The number of genes in the KEGG enriched pathway is indicated by the size of the circle.

**
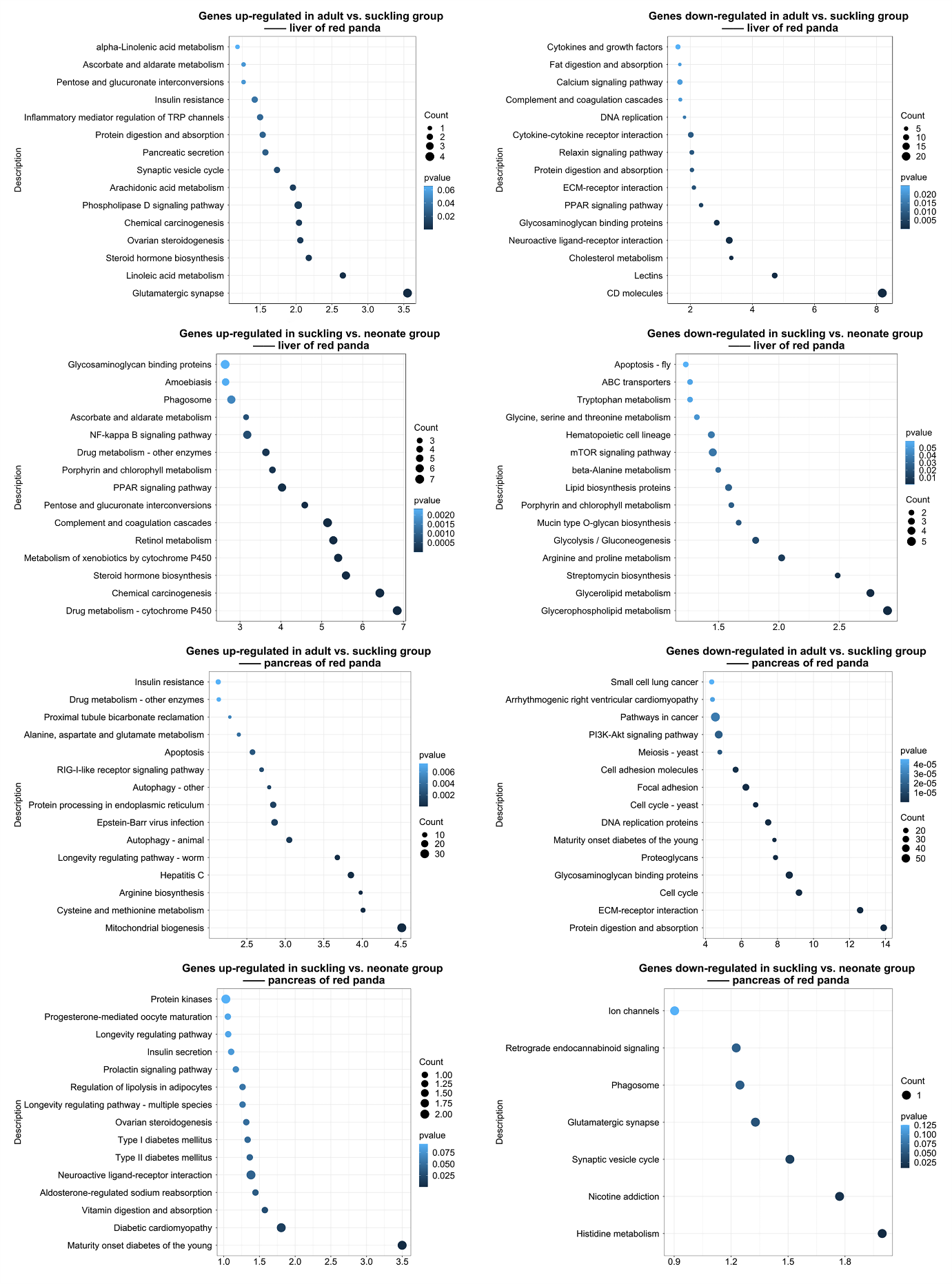
**

**Supplementary Figure 2.** KEGG enrichment results of DEGs in the liver or pancreas of red pandas. X-axis indicates the -log10(P) of KEGG enriched pathways, Y-axis indicates the name of the pathway. The number of genes in the KEGG enriched pathway is indicated by the size of the circle.

**
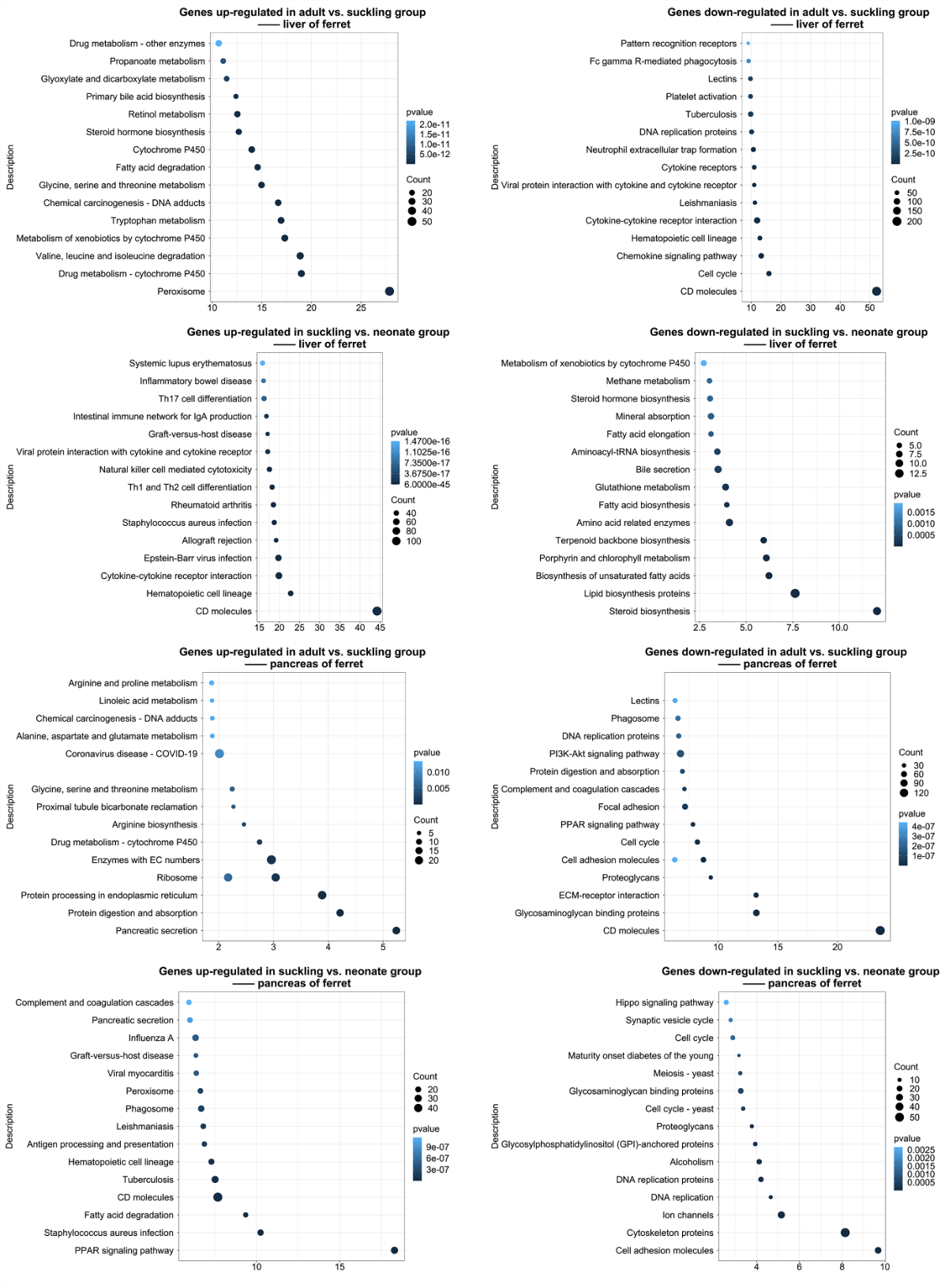
**

**Supplementary Figure 3.** KEGG enrichment results of DEGs in the liver or pancreas of ferrets. X-axis indicates the -log10(P) of KEGG enriched pathways, Y-axis indicates the name of the pathway. The number of genes in the KEGG enriched pathway is indicated by the size of the circle.

**
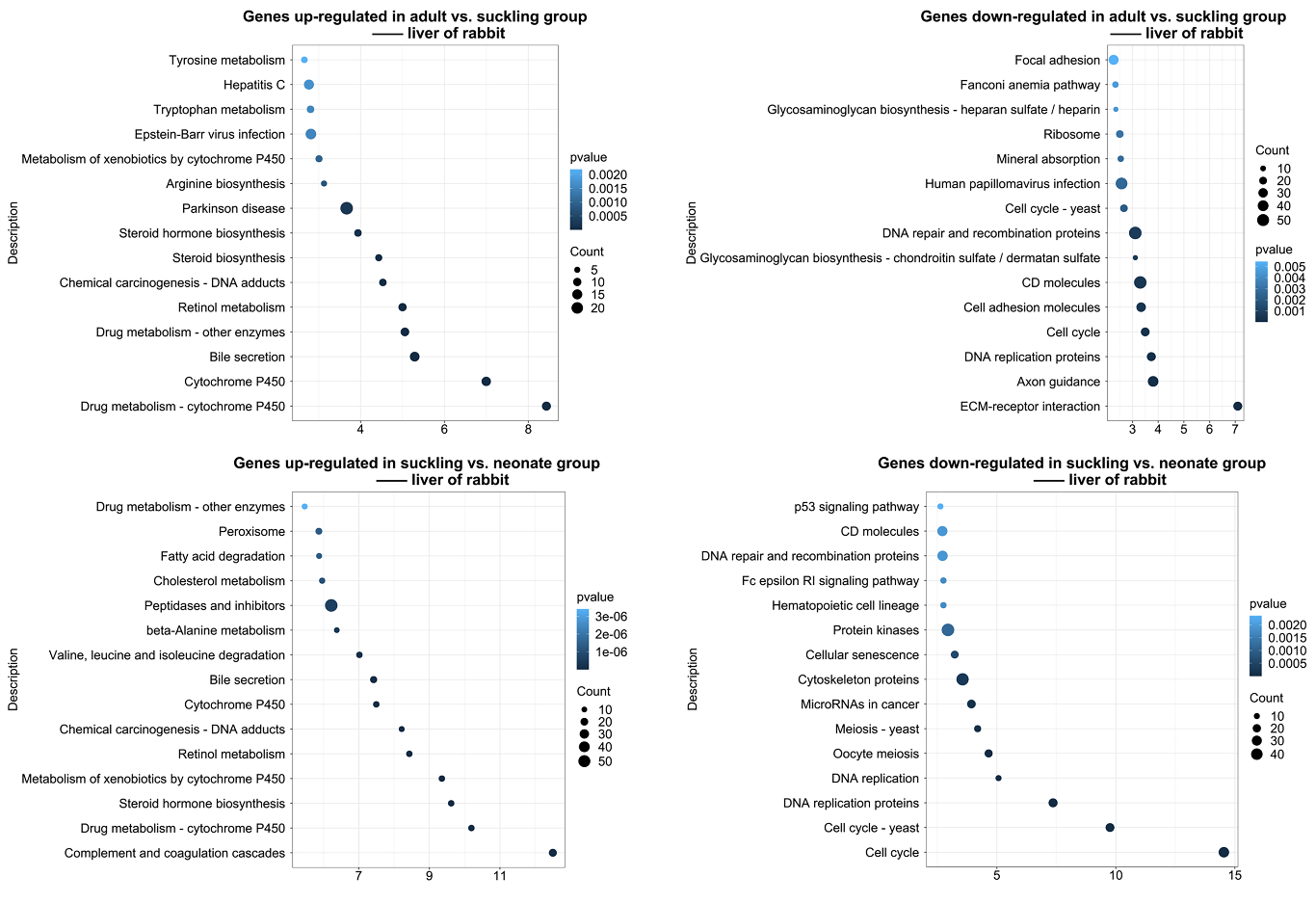
Supplementary Figure 4.** KEGG enrichment results of DEGs in the liver of rabbits. X-axis indicates the -log10(P) of KEGG enriched pathways, Y-axis indicates the name of the pathway. The number of genes in the KEGG enriched pathway is indicated by the size of the circle.


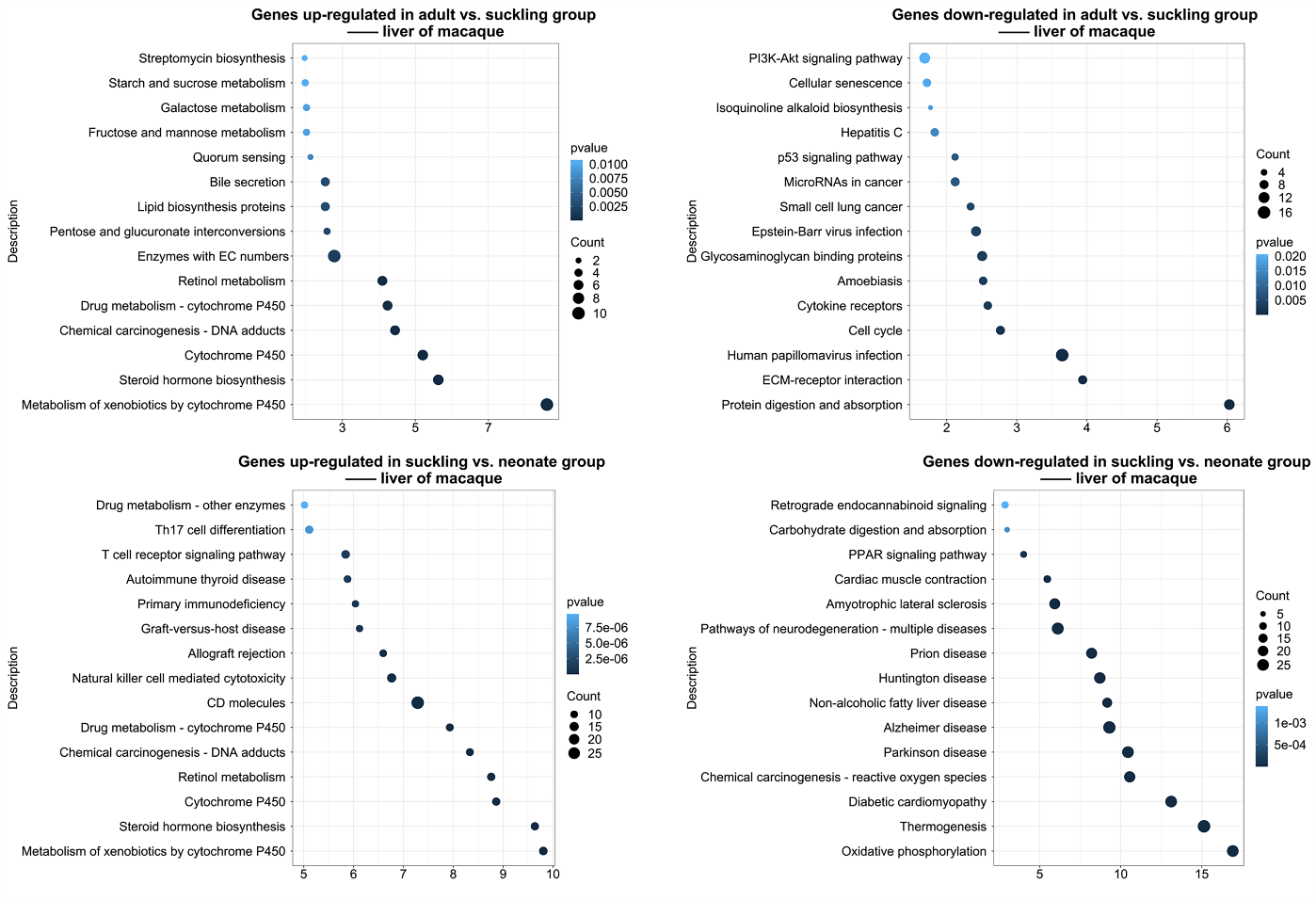


**Supplementary Figure 5.** KEGG enrichment results of DEGs in the liver of macaques. X-axis indicates the -log10(P) of KEGG enriched pathways, Y-axis indicates the name of the pathway. The number of genes in the KEGG enriched pathway is indicated by the size of the circle.

**
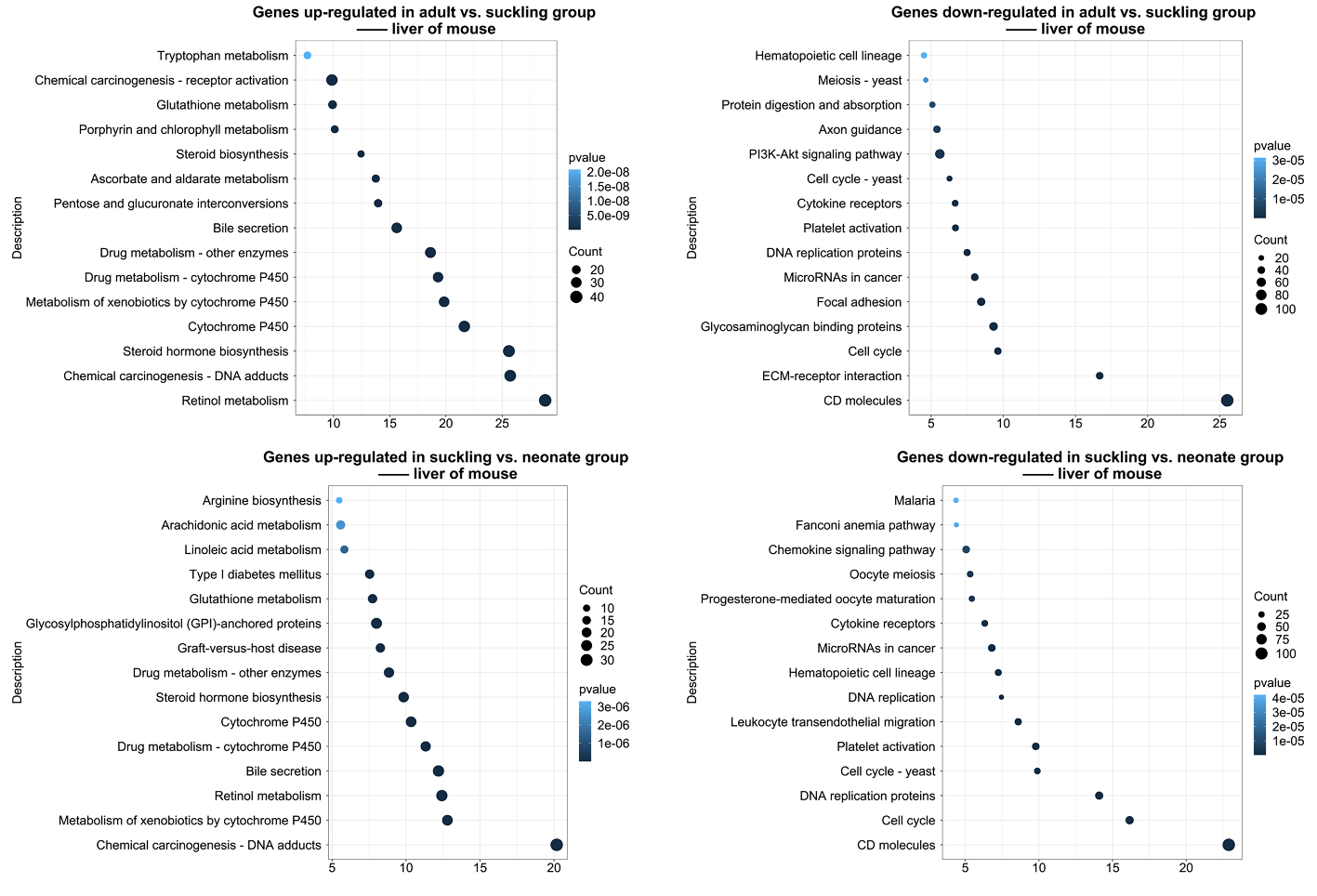
**

**Supplementary Figure 6.** KEGG enrichment results of DEGs in the liver of mice. X-axis indicates the -log10(P) of KEGG enriched pathways, Y-axis indicates the name of the pathway. The number of genes in the KEGG enriched pathway is indicated by the size of the circle.


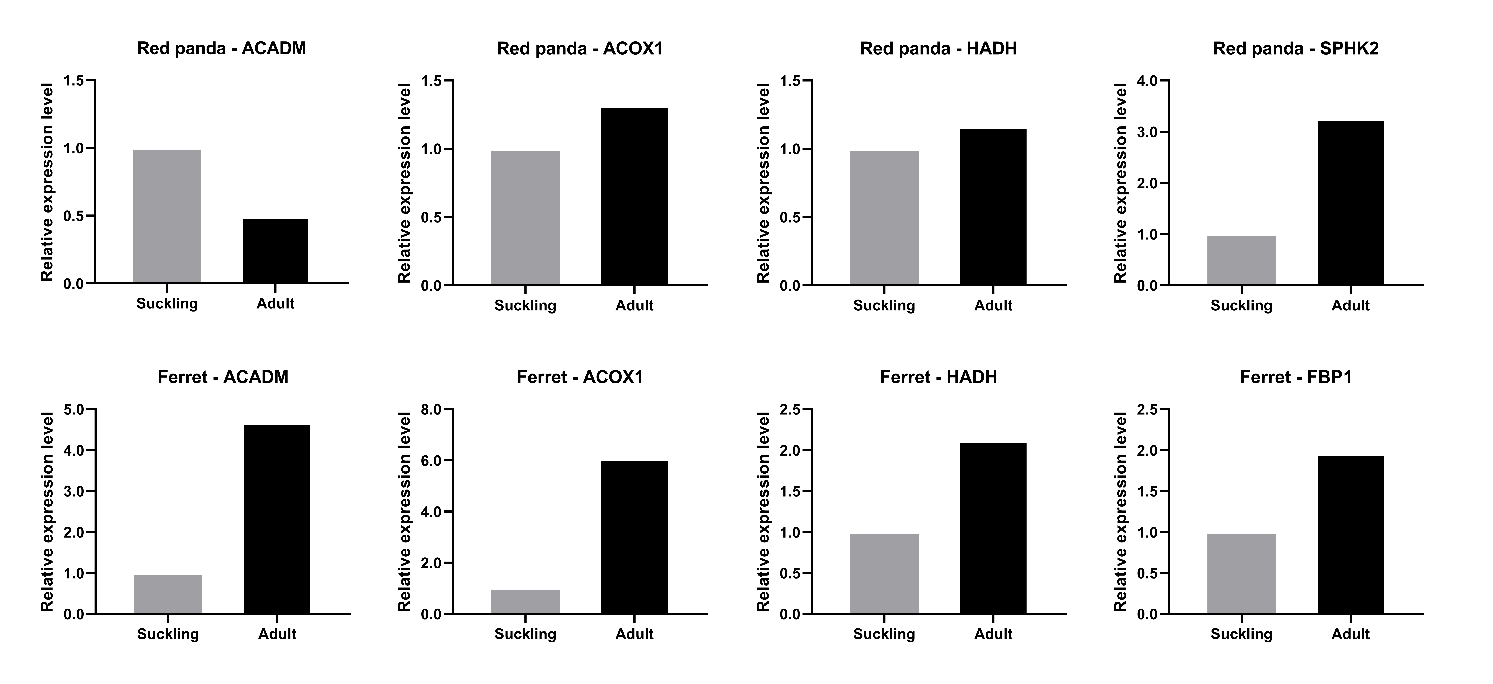


**Supplementary Figure 7.** The expression level of genes calculated by RT-qPCR. Y-axis represents relative expression levels of each gene by using 2^−ΔΔCT^ method.
