# Supplementary figures and images for: Comparative study of the digestion and metabolism related genes’ expression changes during the postnatal food change in different dietary mammals
Source: Front Genet. 2023 Jul 4;14:1198977. doi: 10.3389/fgene.2023.1198977 (PMC10352678; doi:10.3389/fgene.2023.1198977)

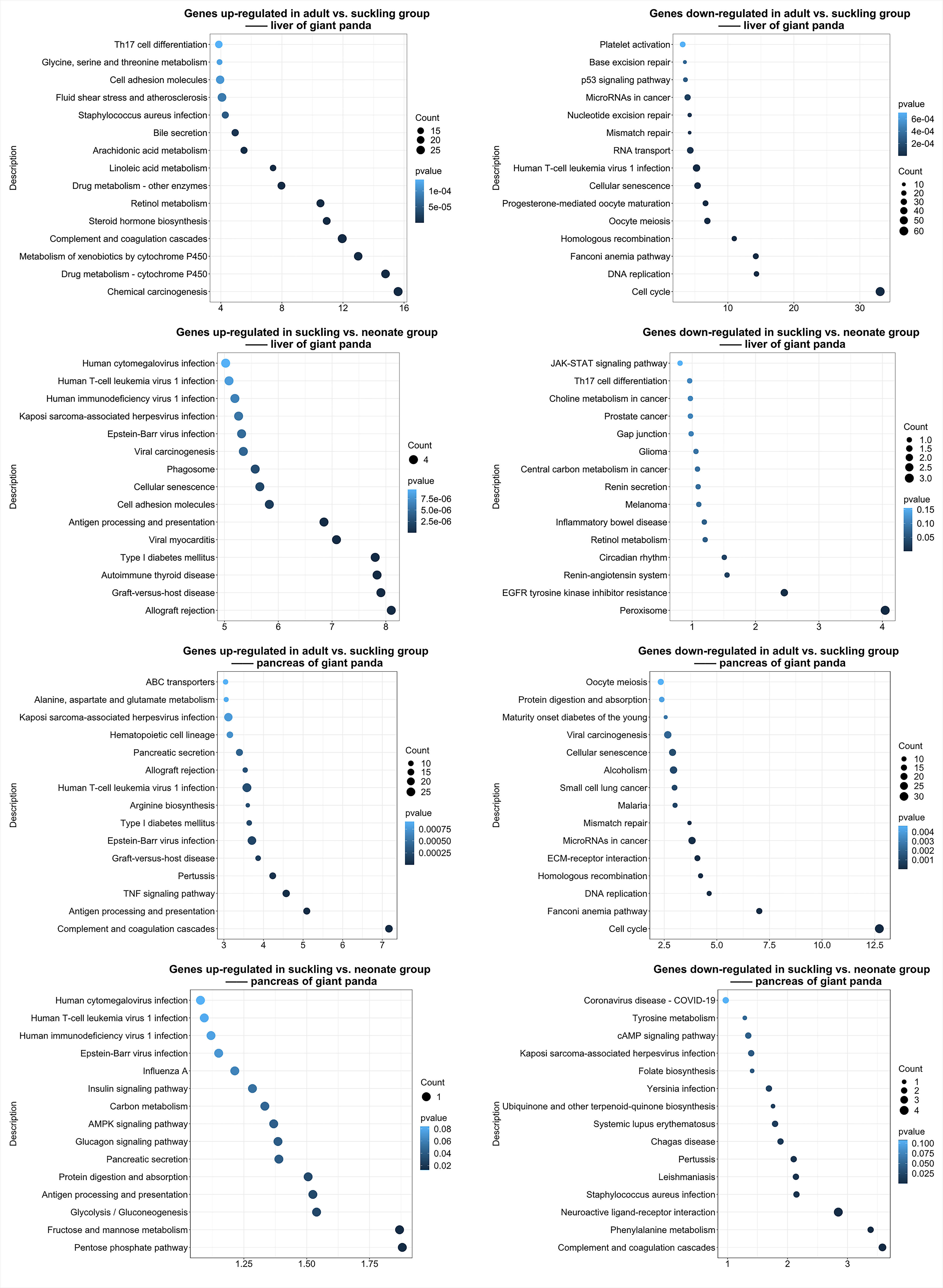

Supplement: Supplementary file 1 [file DataSheet1.zip › Supplementary Figures/Supplementary Figure 1.tif]

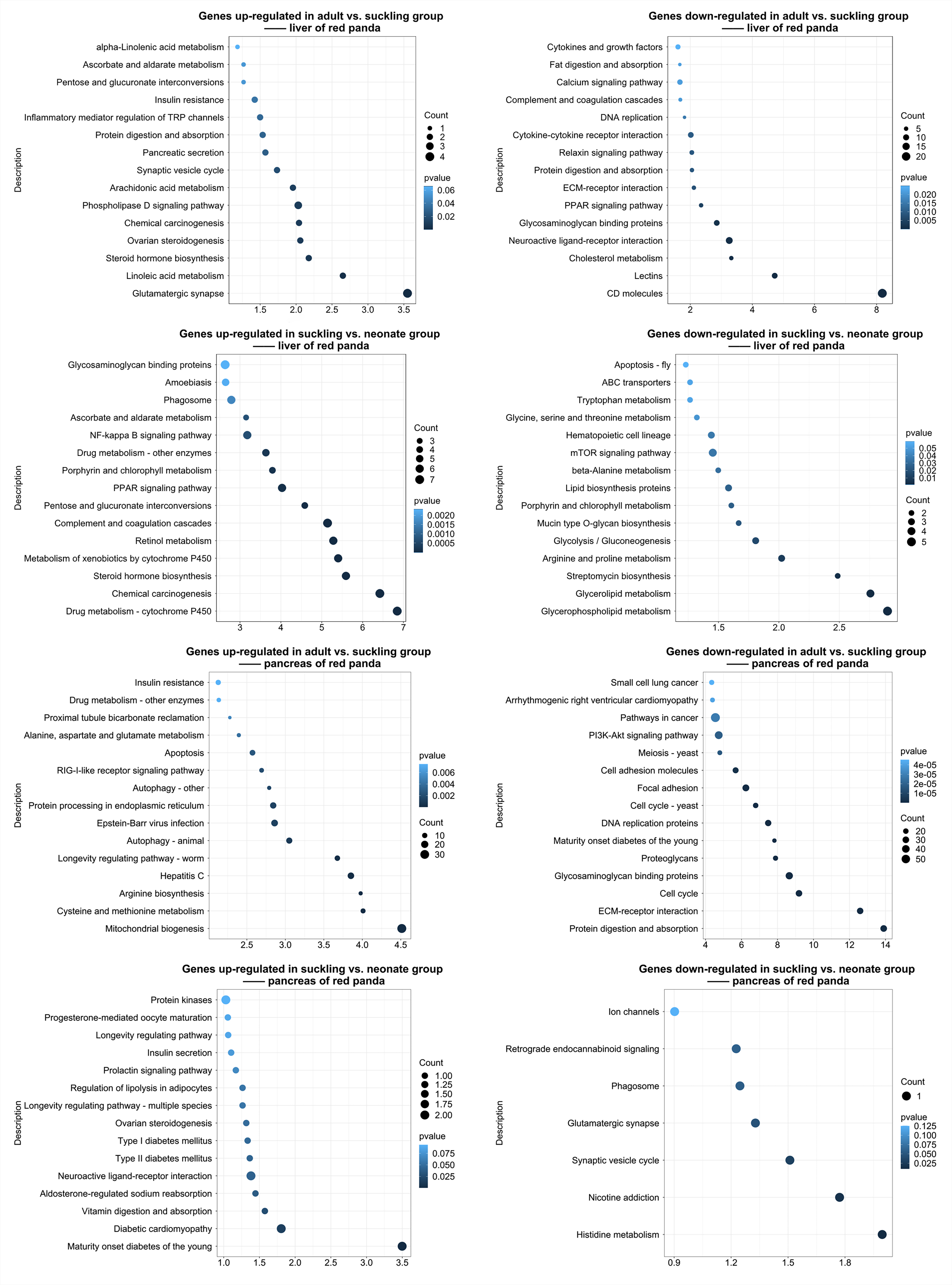

Supplement: Supplementary file 1 [file DataSheet1.zip › Supplementary Figures/Supplementary Figure 2.tif]

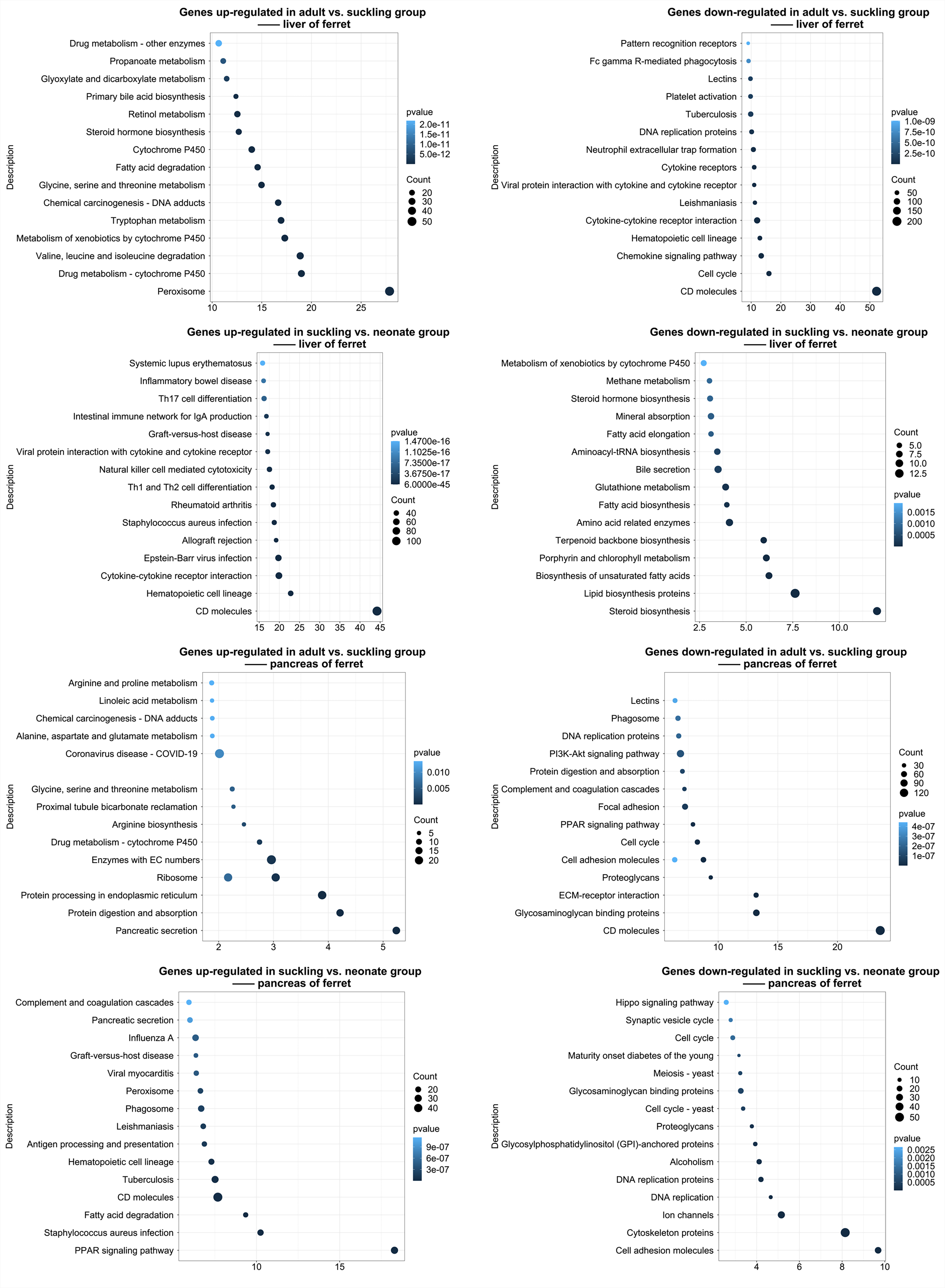

Supplement: Supplementary file 1 [file DataSheet1.zip › Supplementary Figures/Supplementary Figure 3.tif]

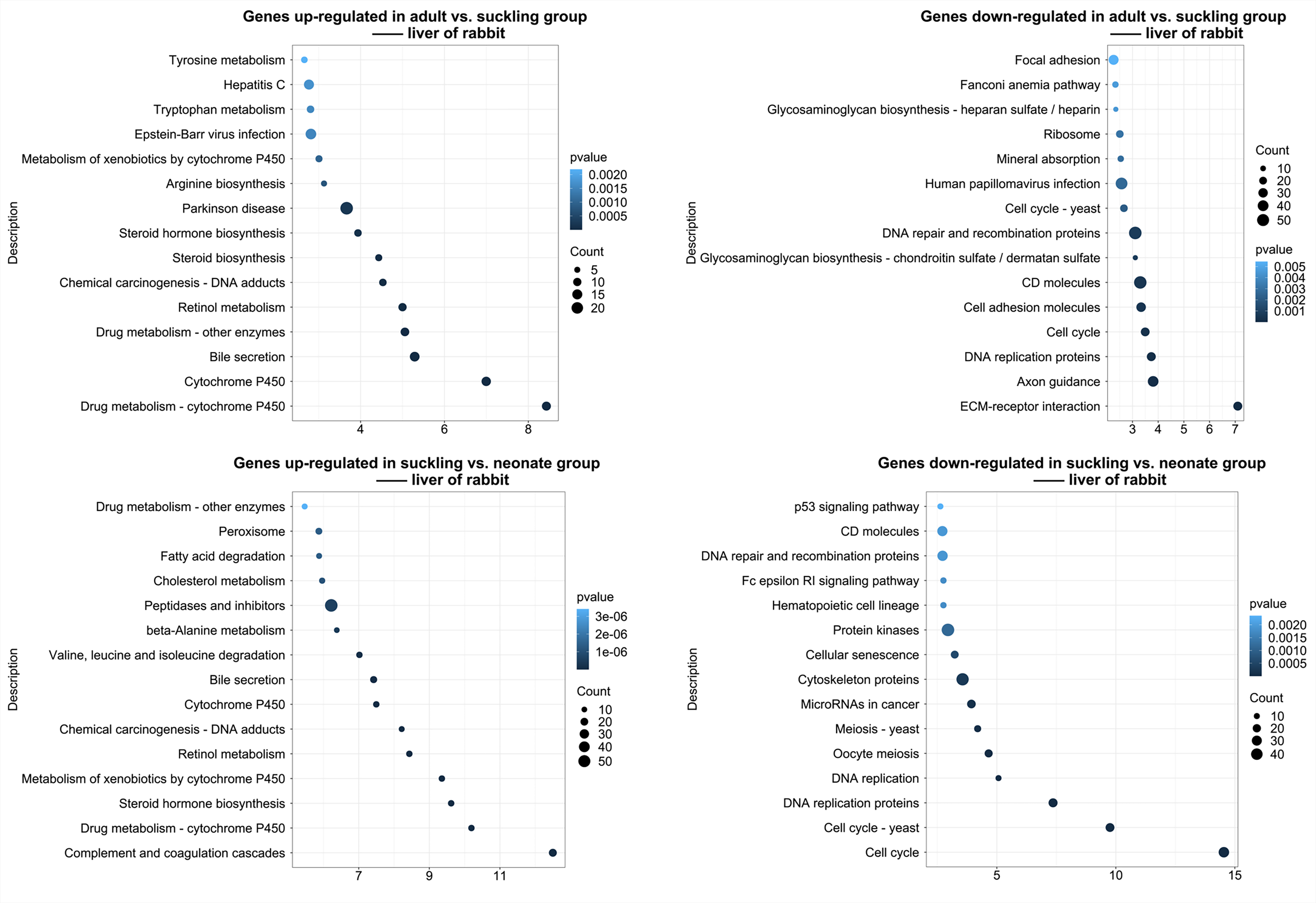

Supplement: Supplementary file 1 [file DataSheet1.zip › Supplementary Figures/Supplementary Figure 4.tif]

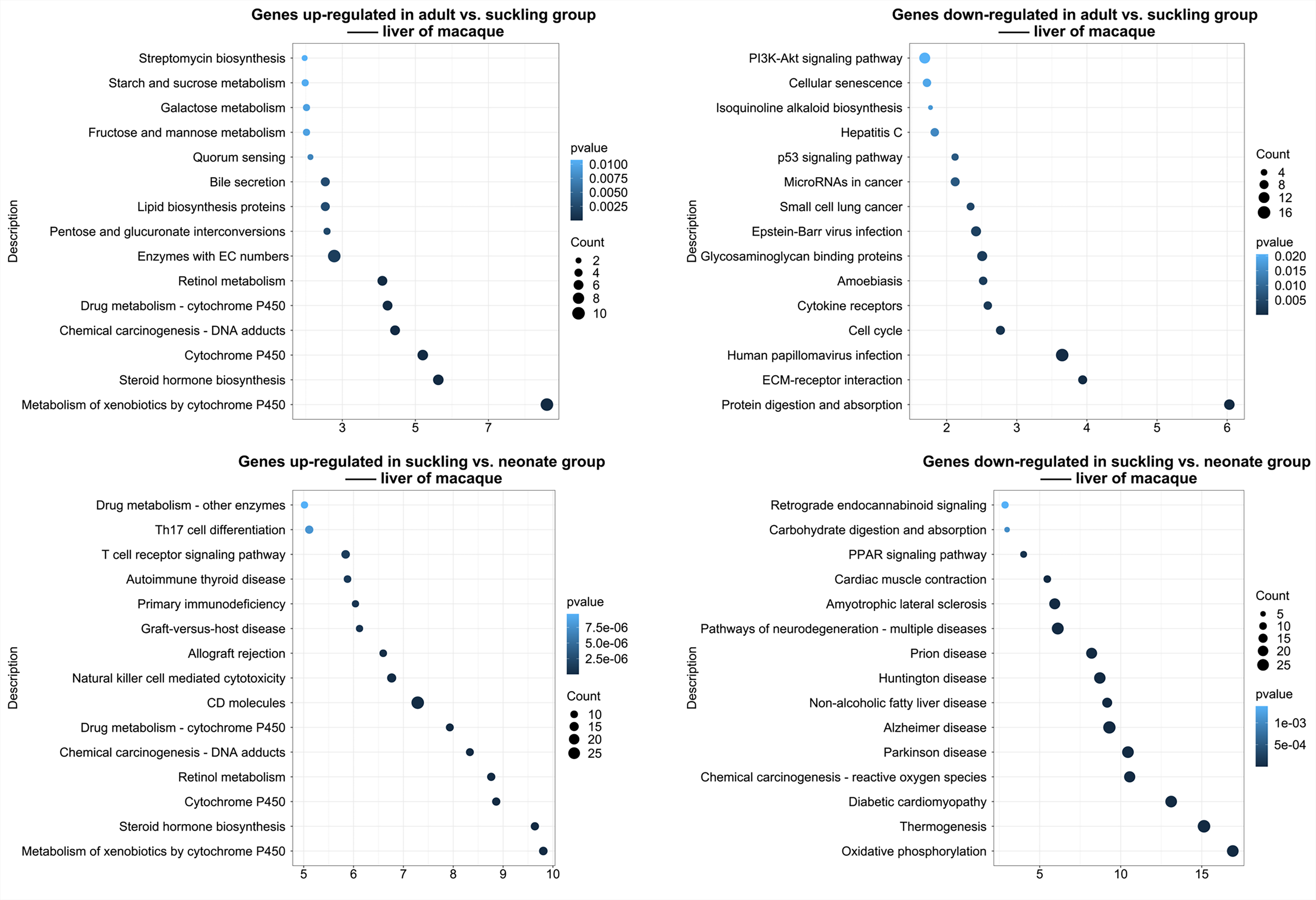

Supplement: Supplementary file 1 [file DataSheet1.zip › Supplementary Figures/Supplementary Figure 5.tif]

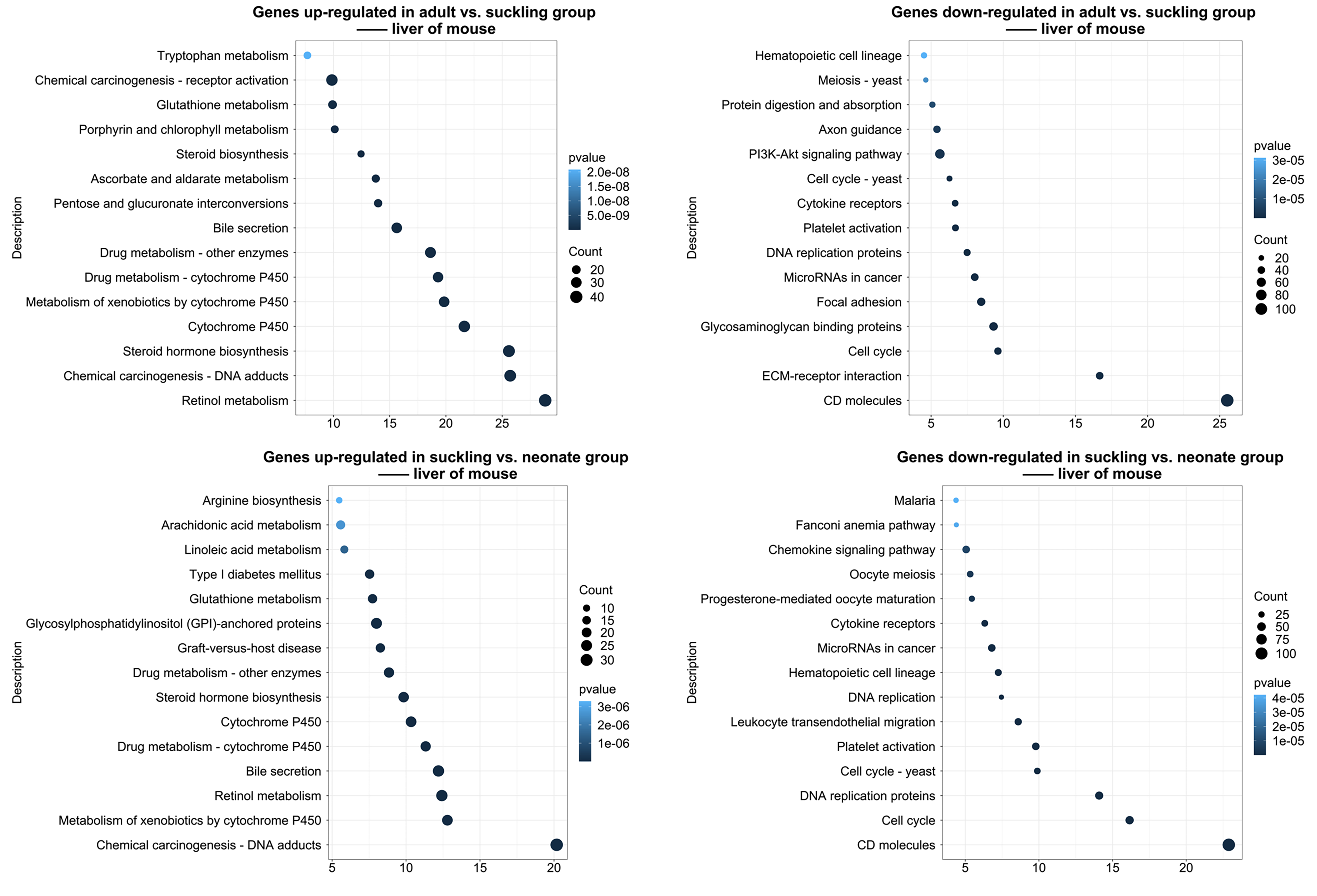

Supplement: Supplementary file 1 [file DataSheet1.zip › Supplementary Figures/Supplementary Figure 6.tif]

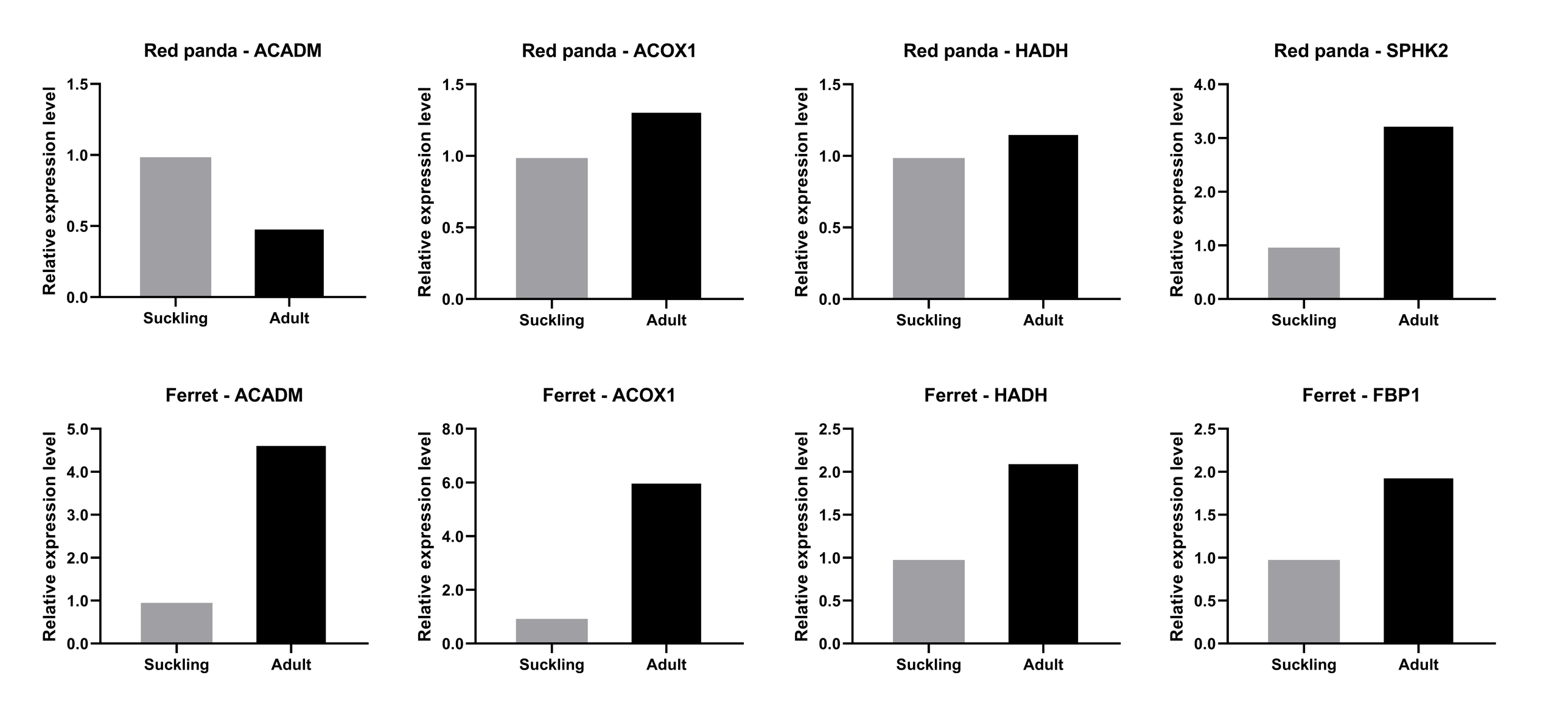

Supplement: Supplementary file 1 [file DataSheet1.zip › Supplementary Figures/Supplementary Figure 7.tif]
